# Investigating the Anti-Inflammatory and Pain-Relieving Properties of *Glaucium corniculatum* (L.) Rud subsp. *refractum* (NAB) Cullen and *Glaucium leiocarpum* Boiss., Along with Their Active Compounds

**DOI:** 10.3390/molecules31152711

**Published:** 2026-08-04

**Authors:** Melek Karaaslan, Burçin Ergene, Hediye Kamuran İleri, Okan Ekim, Özlem Bahadır-Acıkara, Hanefi Özbek, Betül Sever-Yılmaz, Mehmet Levent Altun

**Affiliations:** 1 Department of Pharmacognosy, Faculty of Pharmacy, Ankara University, 06560 Ankara, Türkiye; mkaracaoglu@ankara.edu.tr (M.K.); ergene@pharmacy.ankara.edu.tr (B.E.); ilerik@ankara.edu.tr (H.K.İ.); sever@pharmacy.ankara.edu.tr (B.S.-Y.); altun@pharmacy.ankara.edu.tr (M.L.A.); 2 Department of Veterinary Anatomy, Faculty of Veterinary, Ankara University, 06070 Ankara, Türkiye; ekim@ankara.edu.tr; 3 Department of Pharmacology, Faculty of Medicine, Istanbul Medeniyet University, 34700 İstanbul, Türkiye; hanefi.ozbek@medeniyet.edu.tr

**Keywords:** anti-inflammatory activity, antinociceptive activity, flavonoid, *Glaucium*, *Glaucium corniculatum*, *Glaucium leiocarpum*

## Abstract

The genus *Glaucium* (Papaveraceae), comprising approximately 30 species, is widely distributed across the Mediterranean region, Europe, North Africa, and Central and Southwest Asia. In the flora of Türkiye, the genus is represented by 11 taxa and is commonly known as “boynuzlu gelincik” and “çömlek çatlatan”. The aim of this study is to explore the analgesic and anti-inflammatory properties of *Glaucium corniculatum* (L.) Rud subsp. *refractum* (NAB) Cullen, and *Glaucium leiocarpum* Boiss., which are found in Türkiye and are known for their high alkaloid content. Traditionally, *G. leiocarpum* has been used for wound care, cuts, bruises, acne, hemorrhoids, headache, and diarrhea, while *G. corniculatum* subsp. *refractum* has been employed as an antitussive and for external applications against ocular inflammations. Ethanolic extracts and crude alkaloid fractions from the aerial parts were evaluated in vivo in anti-inflammatory and antinociceptive test models. Anti-inflammatory activity was assessed by the carrageenan-induced paw edema model, while antinociceptive activity was determined using tail-flick and hot-plate tests. *G. leiocarpum* ethanolic extract (200 mg/kg) showed the highest anti-inflammatory activity, effectively suppressing carrageenan-induced edema (1.58 ± 0.30–1.73 ± 0.51 mL), and displayed the strongest antinociceptive activity, with tail-flick latencies reaching 23.91 ± 2.08 s (90 min) and 22.23 ± 1.97 s (150 min) and hot-plate latencies of 22.27 ± 4.76 s (30 min) and 23.00 ± 4.61 s (90 min). Bioactivity-guided fractionation of the most active extract was carried out using various chromatographic techniques. Thin-layer chromatography (TLC) and high-performance liquid chromatography (HPLC) analyses were employed throughout the isolation process to monitor fractions and determine their phytochemical profiles. As a result, two compounds were obtained and subsequently evaluated for their biological activities. The chemical structures of the isolated compounds were elucidated by spectroscopic methods, including ^1^H-NMR, ^13^C-NMR, 2D-NMR, and LC-MS analyses as kaempferol-3-*O*-glucorhamnoside and quercetin-3-*O*-glucorhamnoside. Biological activity evaluation demonstrated that these compounds significantly contributed to the observed antinociceptive activity. These findings confirm that *Glaucium* species are traditionally used and highlight their potential to provide bioactive compounds with anti-inflammatory and pain-relieving effects.

## 1. Introduction

Medicinal plants represent a valuable source of bioactive compounds with potential therapeutic applications, and their use continues to gain scientific and clinical relevance [1]. *Glaucium* species, which belong to the Papaveraceae family, have been utilized for various therapeutic purposes in folk medicine across different regions worldwide [2]. Their applications include the treatment of respiratory illnesses and their use as laxatives, sedatives, antidiabetic agents, and remedies for dermatitis [3]. Traditionally, the aerial parts of the plant were employed as narcotics or hypnotics, while the seeds were historically used as laxatives [4]. The aerial parts of *Glaucium leiocarpum* Boiss are mixed with honey and used in the form of a plaster for wound care, cuts, bruises, stretch marks, acne and hemorrhoids. Its leaves are used externally, either alone or mixed with tobacco, to treat headaches. It has also been reported that its flowers are crushed with olive oil when fresh and used externally for wound healing and internally as a decoction in the treatment of diarrhea [5,6,7,8,9]. Similarly, *Glaucium corniculatum* (L.) Rudolph subsp. *refractum* (NAB) Cullen has traditionally been used as an antitussive, and its leaves and fruits have been applied externally for the treatment of ocular inflammation [10,11]. Despite these widespread traditional uses, the pharmacological activities of these species, particularly their anti-inflammatory and antinociceptive effects, remain insufficiently explored.

Given the increasing interest in validating traditional medicinal practices through scientific approaches, the present study aimed to investigate the anti-inflammatory and antinociceptive activities of *G. leiocarpum* and *G. corniculatum* subsp. *refractum* using established in vivo models. Furthermore, a bioactivity-guided fractionation approach was employed to isolate and characterize the compounds responsible for the observed biological activities. Within the scope of our project, a carrageenan-induced paw edema test was performed in rats to determine acute anti-inflammatory activity. For the determination of antinociceptive activity, the tail-flick test and the hot-plate test were used.

## 2. Results

### 2.1. Phytochemical Analysis Results

**Figure 1:**
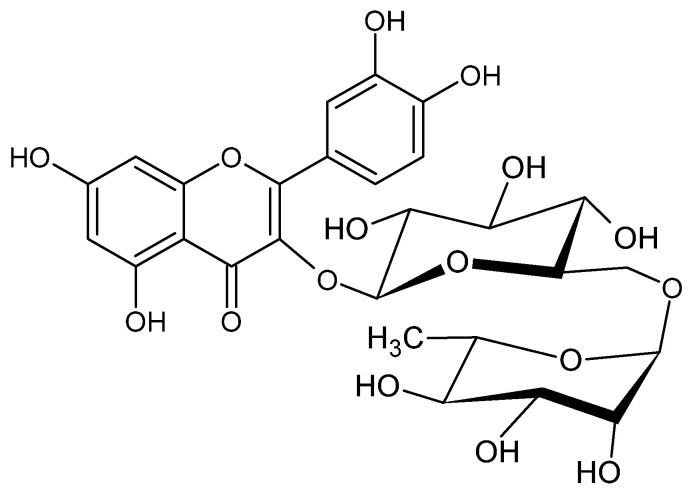
Chemical structure of compound A.

**Figure 2:**
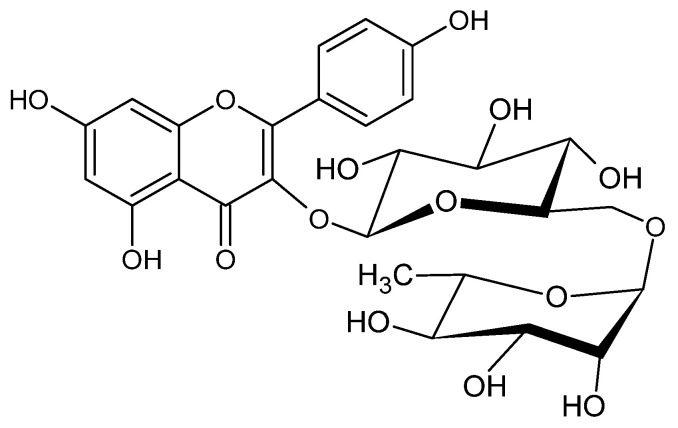
Chemical structure of compound B.

GLT was identified as the most promising extract among all *Glaucium* ethanolic and total alkaloid extracts tested. Therefore, the ethanolic extract of GLT was subjected to liquid–liquid fractionation to obtain fractions and active compounds. The ethyl acetate fraction, which showed the highest activity in the tail-flick and hot-plate tests, was chromatographed on a silica gel column to identify the compounds responsible for the antinociceptive activity. Elution was performed using the appropriate solvent system, which resulted in the yield of subfractions labelled A1–A6. These subfractions were tested for anti-inflammatory and antinociceptive activity, with A4 showing the greatest activity. Following column chromatography on a silica gel column and preparative TLC, the compounds were isolated and named compound A (8 mg) and compound B (4.7 mg). Their structures were elucidated using spectroscopic techniques, including NMR and MS. Compound A was identified as quercetin-3-*O*-glucorhamnoside (Figure 1) and compound B as kaempferol-3-*O*-glucorhamnoside (Figure 2).

Compound A, ^1^H NMR (400 MHz, DMSO-d6) δ: 7.56 (1H, br s, H-2′), 7.54 (1H, dd, *J* = 8.0, 2.0 Hz, H-6′), 6.76 (1H, d, *J* = 8.0 Hz, H-5′), 6.21 (1H, d, *J* = 2.0 Hz, H-6), 6.04 (1H, d, *J* = 2.0 Hz, H-8), 4.94 (1H, d, *J* = 10.0 Hz, H-1″), 4.43 (1H, br s, H-1‴), 1.03 (3H, d, *J* = 6.0 Hz, H-6‴).

Compound B, ^1^H NMR (400 MHz, DMSO-d6) δ: 8.08 (2H, d, *J* = 8.0 Hz, H-2′, H-6′), 6.90 (2H, d, *J* = 8.0 Hz, H-3′, H-5′), 6.35 (1H, d, *J* = 2.0 Hz, H-6), 6.17 (1H, d, *J* = 2.0 Hz, H-8), 5.09 (1H, d, *J* = 10.0 Hz, H-1″), 4.53 (1H, br s, H-1‴), 1.14 (3H, d, *J* = 6.0 Hz, H-6‴).

### 2.2. Biological Activity Test Results

#### 2.2.1. Anti-Inflammatory Activity

This research study aimed to evaluate the anti-inflammatory properties of several extracts obtained from *G. leiocarpum* (GL) and *G. corniculatum* subsp. *refractum* (GC), offering a comprehensive analysis of their effects on inflammation. The focus is on ethanolic extracts, classified as GLT and GCT, as well as on total alkaloid extracts obtained from the same plant species, named GLA and GCA. Anti-inflammatory activity results are summarized in Table 1, which collectively illustrates the varying degrees of anti-inflammatory activity exhibited by each extract. In the carrageenan-induced paw edema model, the control group exhibited a clear and progressive increase in paw volume, rising from baseline (0 h; 1.67 ± 0.16 mL) to the sixth hour (2.46 ± 0.51 mL). Statistically significant differences were observed at all time points compared to baseline values (*p* < 0.05), confirming successful induction of inflammation. The reference drug indomethacin significantly suppressed edema formation during the early phase of inflammation, with paw volumes remaining relatively low between the first hour (1.54 ± 0.14 mL) and the third hour (1.67 ± 0.19 mL). However, significant increases became evident from the third hour onward (*p* < 0.05), indicating a time-dependent decline in its anti-inflammatory effect.

Among the tested extracts, GLT administered at 200 mg/kg exhibited the most pronounced anti-inflammatory activity. Paw volumes remained relatively stable between the first hour (1.58 ± 0.30 mL) and the sixth hour (1.73 ± 0.51 mL), with no statistically significant increase observed between the second and sixth hours, suggesting effective inhibition of edema formation during the late inflammatory phase. In contrast, GCT administered at 200 mg/kg showed limited activity, with paw volume increasing steadily from baseline (0 h; 1.65 ± 0.07 mL) to the sixth hour (1.95 ± 0.29 mL). At the dose of 100 mg/kg, both GLT and GCT demonstrated moderate anti-inflammatory effects. GLT at 100 mg/kg showed a gradual increase in paw volume through the sixth hour, with statistically significant differences becoming apparent from the second hour onward. Similarly, GCT at 100 mg/kg increased from 1.53 ± 0.05 mL at baseline to 2.04 ± 0.13 mL at the sixth hour, indicating only slight and limited inhibition of carrageenan-induced inflammation. At the 50 mg/kg dose, both GCT and GLT extracts exhibited weaker activity, resulting in insufficient suppression of edema formation.

The alkaloid fractions GCA and GLA exhibited only weak anti-inflammatory activity in the carrageenan-induced paw edema model. At 100 mg/kg, paw volume in the GCA-treated group increased from 1.75 ± 0.19 mL at baseline to 2.24 ± 0.22 mL at the sixth hour, reflecting a progression of inflammation comparable to that of the control group. Similarly, the GLA fraction showed only limited activity, with paw volume increasing from 1.57 ± 0.09 mL to 2.07 ± 0.18 mL over the same period. Both the GCA and GLA fractions administered at a dosage of 50 mg/kg demonstrated effects comparable to those observed with the 100 mg/kg treatment. However, none of the doses exhibited significant suppression of carrageenan-induced inflammation, as detailed in Table 1.

Overall, the findings demonstrated a clear dose-dependent anti-inflammatory effect, with the GLT extract at 200 mg/kg exhibiting the most potent inhibitory activity, particularly during the late phase of carrageenan-induced inflammation (2–6 h). Owing to its pronounced activity profile, the GLT extract was selected for further phytochemical and biological investigations.

In addition to the total ethanolic extracts, the anti-inflammatory activities of three fractions obtained from the GLT extract were also evaluated. These fractions consisted of petroleum ether (GLT-PE), ethyl acetate (GLT-EtOAc), and water (GLT-water), prepared by liquid–liquid partitioning of the total extract. Table 1 also presents a detailed comparison of fractions. The GLT-EtOAc fraction was found to exhibit the highest anti-inflammatory activity among the tested fractions. Paw volumes remained relatively stable throughout the experimental period, increasing only slightly from 1.46 ± 0.18 mL at baseline (0 h) to 1.59 ± 0.11 mL at the fourth hour and 1.60 ± 0.10 mL at the fifth hour. At the sixth hour, paw volume reached 1.65 ± 0.11 mL, which remained markedly lower than that of the control group (2.46 ± 0.51 mL). Only limited statistically significant increases were observed during the observation period, indicating a strong inhibitory effect on carrageenan-induced edema formation. In contrast, the GLT-PE fraction exhibited minimal anti-inflammatory activity. Paw volumes increased progressively from baseline to the sixth hour, following a pattern comparable to that of the control group, and no marked inhibition of edema development was observed. The water fraction showed moderate but inconsistent activity. Paw volume increased from 1.53 ± 0.21 mL at baseline to 1.89 ± 0.35 mL at the sixth hour. Although occasional statistically significant differences were detected at certain time points, the overall effect remained limited, suggesting relatively weak anti-inflammatory potential compared with that of the GLT-EtOAc fraction.

Table 1 presents the fractions obtained from the GLT-EtOAc phase by chromatographic techniques to identify the compounds responsible for anti-inflammatory activity. Subsequent fractionation of the GLT–EtOAc extract indicated distinct activity profiles across the various fractions. The fractions A1 and A2 were tested together in animals, but they did not exhibit significant anti-inflammatory effects. Paw volume gradually increased from 1.59 ± 0.08 mL at 0 h to 2.14 ± 0.16 mL at the sixth hour, and these changes were statistically significant (*p* < 0.001), suggesting no inhibition of carrageenan-induced edema. Fraction A3 showed only weak effects. The paw volume changed from 1.50 ± 0.07 mL at the start to 1.64 ± 0.12 mL after 6 h. The differences were small and inconsistent, which indicates a limited ability to reduce swelling. The rise in paw volumes was observed, increasing from 1.59 ± 0.12 mL at the 0th time point to 2.01 ± 0.24 mL at the sixth time point following treatment with fractions A5-A6, indicating that these fractions exhibit minor activity against inflammation. On the other hand, fraction A4 displayed the most pronounced anti-inflammatory effect among the tested fractions. Throughout the study, paw volumes remained relatively stable. They changed from 1.45 ± 0.05 mL at the start to 1.63 ± 0.32 mL at the fifth hour and 1.71 ± 0.34 mL at the sixth hour. There were no significant differences between the second and sixth hours. This suggests that A4 significantly reduced swelling, especially during the late phase of inflammation. Taken together, these findings suggest that fraction A4 represents the most promising fraction for further phytochemical isolation and characterization studies.

**Figure 3:**
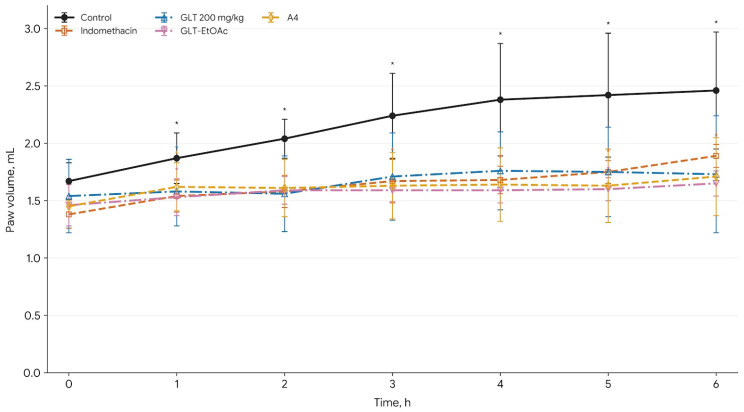
Anti-inflammatory activity of the most active treatment groups in the carrageenan-induced paw edema model. Complete datasets for all experimental groups are provided in Table 1. Values are expressed as mean ± SD (*n* = 6). Statistical comparisons were performed between right and left paws (paired analysis).* *p* < 0.05: statistically significant difference. No symbol: not significant (*p* > 0.05).

For clarity, the anti-inflammatory responses of the most active treatment groups are graphically presented in Figure 3.

#### 2.2.2. Antinociceptive Activity

##### Tail-Flick Test

The antinociceptive activity of extracts from *Glaucium* species was assessed using the tail-flick test, and the results are outlined in Table 2. The control group (CMC) did not show any change in pain response during the experiment, while the reference drug, morphine, significantly increased the time to tail flick, reaching its maximum effect at 60 min and maintaining a significant effect at 90 min (*p* < 0.05 compared to control), confirming its known pain-relieving properties. GLT at 200 mg/kg had the strongest effect, showing a steady increase in the pain response over time compared with other extracts, whereas GCT at 200 mg/kg showed moderate activity, although the effects were not statistically significant compared with the control group. The highest response was observed at 90 and 150 min, with tail-flick latencies of 23.91 ± 2.08 s and 22.23 ± 1.97 s, respectively, with the GLT treatment at a dose of 200 mg/kg. Lower doses (100 and 50 mg/kg) of both GLT and GCT demonstrated minimal or no significant effects at all tested time points.

Interestingly, GLA at 50 mg/kg displayed a significant increase in latency at 150 min (21.26 ± 3.06 s), (*p* < 0.01), suggesting a delayed but notable antinociceptive effect. Other groups, including GLA at 100 mg/kg and GCA extracts, showed moderate increases that did not induce statistical significance.

Table 2 also displays the tail-flick latency after treatment with the GLT phases. GLT-EtOAc demonstrated the most prominent and consistent antinociceptive activity. Latency times increased from 9.97 ± 3.00 s at baseline to 17.57 ± 4.70 s at 60 min and 20.28 ± 6.74 s at 90 min (*p* < 0.01), reaching a maximum of 21.00 ± 7.80 s at 150 min (*p* < 0.001). Significant increases in latency were observed at 60 and 90 min (*p* < 0.01), with a further marked effect at 150 min (*p* < 0.001). These results indicate that the active constituents responsible for the analgesic effect are predominantly concentrated in the semi-polar fraction. Notably, the effects observed at 90 and 150 min were comparable to, and at later time points even exceeded, those of morphine, indicating a strong and sustained central analgesic effect.

In contrast, GLT-PE showed only slight increases in latency (e.g., 12.28 ± 2.56 s at 60 min), which were not statistically significant. Similarly, GLT-water did not produce meaningful changes in latency times across all time points (e.g., 10.85 ± 2.43 s at 90 min; 10.27 ± 1.16 s at 150 min), indicating negligible antinociceptive activity.

The antinociceptive effects of the fractions (A1–A6) were evaluated by measuring tail-flick latency time at 0, 30, 60, 90, and 150 min following administration. As shown in Table 2, among the samples tested, subfraction A4 was the most effective, with an average value of 18.88 ± 6.09 s, which was close to the reference drug’s activity (15.99 ± 7.62 s).

The other fractions showed moderate or low effects. After 60 min, the difference in activity remained statistically significant. The reference (morphine) group had the highest response (20.13 ± 5.12 s), followed by A5 (16.10 ± 6.97 s) and A4 (14.75 ± 7.73 s), indicating that these fractions had lasting effects. After 90 min, significant differences were observed between A4 (15.33 ± 3.10 s) and A3 (14.83 ± 1.55 s), indicating strong activity, whereas A6 displayed lower activity (10.83 ± 1.87 s). The reference group maintained a strong response (17.92 ± 4.38 s). On the other hand, at 150 min, there were no statistically significant differences among the groups, suggesting that the effects either declined or became similar over time.

**Figure 4:**
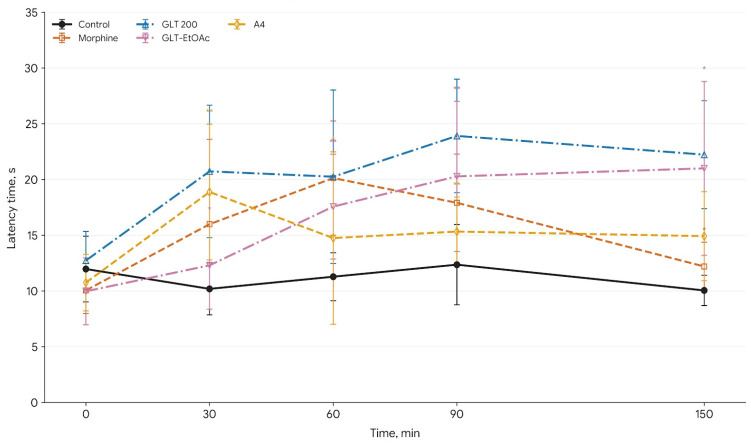
Antinociceptive activity in the tail-flick test. Only the most active treatment groups are shown for clarity; complete datasets for all experimental groups are provided in Table 2. Values are expressed as mean ± SD (*n* = 6). Statistical analysis was performed using one-way ANOVA followed by appropriate post hoc test. Significance levels indicate comparison versus control group: * *p* < 0.05, ** *p* < 0.01 versus baseline (0 min) within the same group (Bonferroni post hoc test). Latency times are expressed in seconds (s).

The antinociceptive responses of the most active treatment groups in the tail-flick test are illustrated in Figure 4 for clarity.

**Figure 5:**
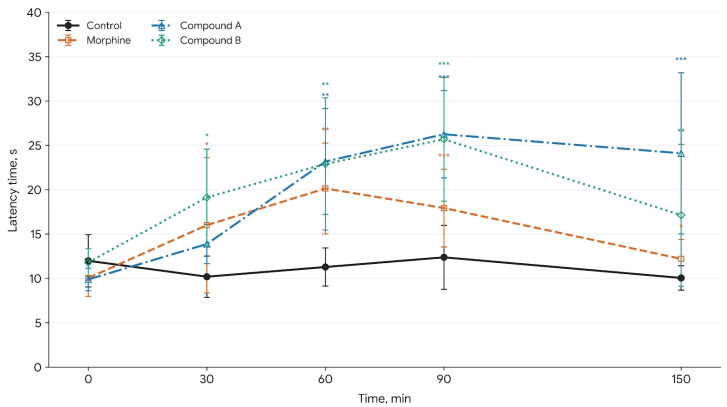
Antinociceptive activity of the isolated compounds in the tail-flick test. Complete datasets for the isolated compounds are provided in Table 3. Values are expressed as mean ± SD (*n* = 6). Statistical analysis was performed using one-way ANOVA followed by appropriate post hoc test. Significance levels indicate comparison versus control group: * *p* < 0.05, ** *p* < 0.01, *** *p* < 0.001 versus baseline (0 min) within the same group (Bonferroni post hoc test). Latency times are expressed in seconds (s).

Table 3 displays the tail-flick test results of the isolated compounds. Compound A displayed the most significant antinociceptive effects. A notable increase in latency time was observed at 60 min (23.17 ± 5.97 s, *p* < 0.01), peaking at 90 min (26.25 ± 4.93 s, *p* < 0.001) and remaining significantly elevated at 150 min (24.10 ± 9.09 s, *p* < 0.001). Compound B also showed considerable antinociceptive effects, with an initial response at 30 min (19.12 ± 5.46 s, *p* < 0.05), followed by significant increases at 60 min (22.90 ± 7.46 s, *p* < 0.01), 90 min (25.68 ± 6.98 s, *p* < 0.001), and 150 min (17.12 ± 8.00 s, *p* < 0.01). While its efficacy was somewhat lower than that of compound A at later time points, compound B still demonstrated a robust and prolonged analgesic effect. The time-dependent antinociceptive effects of the isolated compounds are illustrated in Figure 5.

##### Hot-Plate Test

The morphine reference group showed a significant and rapid increase in latency time, reaching 26.77 ± 4.40 s at 30 min and 25.95 ± 4.13 s at 60 min, followed by a sustained but gradually decreasing effect at 90 min (22.74 ± 3.14 s) and 150 min (18.76 ± 4.88 s) in the hot-plate test. GLT at a dose of 200 mg/kg demonstrated a strong antinociceptive effect, with significant increases at 30 min (22.27 ± 4.76 s) and 90 min (23.00 ± 4.61 s), while showing activity at 60 min (19.53 ± 2.64 s). However, its effect was reduced at 150 min (14.21 ± 2.02 s), indicating a shorter duration compared to the reference.

GCT at 200 mg/kg exhibited moderate yet consistent activity across all time intervals, with latencies of 19.77 ± 4.35 s (30 min), 20.27 ± 6.05 s (60 min), and 20.55 ± 4.75 s (90 min), reflecting a stable but less potent antinociceptive profile. At reduced doses, GLT at 100 mg/kg displayed a delayed onset of action, achieving significance only at 90 min (20.47 ± 5.42 s), while GLT at 50 mg/kg did not show any significant activity at any time point. Similarly, GCT at both 100 mg/kg and 50 mg/kg did not yield significant antinociceptive effects.

The GLA and GCA groups showed time-varying responses. GLA at 100 mg/kg demonstrated significant activity at 60 min (22.95 ± 7.09 s), while GLA at 50 mg/kg showed a delayed but significant effect at 150 min (19.58 ± 5.34 s). GCA at 50 mg/kg exhibited notable activity in the late phase, with latency increasing to 20.32 ± 6.00 s at 90 min (p < 0.05) and to 22.99 ± 2.08 s at 150 min, indicating a prolonged antinociceptive effect. These results are displayed in Table 4.

**Figure 6:**
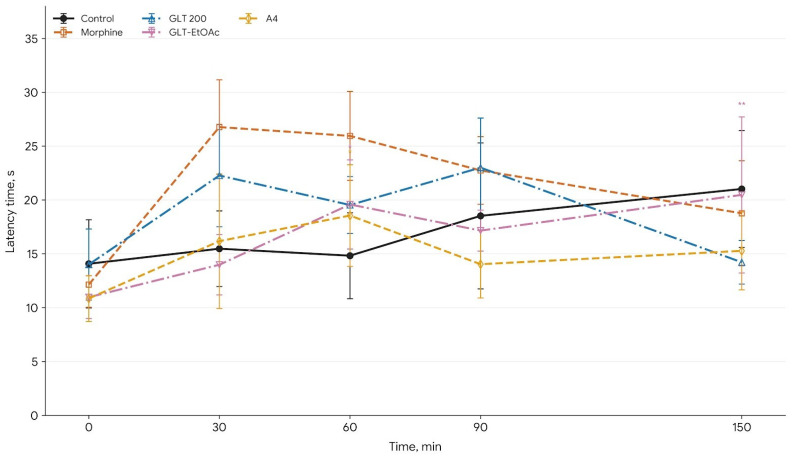
Antinociceptive activity in the hot-plate test. Only the most active treatment groups are shown for clarity; complete datasets for all experimental groups are provided in Table 4. Values are expressed as mean ± SD (*n* = 6). Latency times are expressed in seconds (s). ** *p* < 0.01 versus the control group.

The antinociceptive responses of the most active treatment groups in the hot-plate test are illustrated in Figure 6 for clarity.

GLT-EtOAc among the tested phases demonstrated the most notable activity. A clear increase in latency time was observed at 60 min (19.58 ± 4.14 s), which remained elevated at 90 min (17.15 ± 1.90 s) and further increased at 150 min (20.47 ± 7.25 s), indicating a sustained antinociceptive effect, particularly in the late phase. In contrast, GLT-PE showed minimal activity across all time points, with latency values remaining relatively low (13.97 ± 2.23 s at 60 min and 11.03 ± 3.37 s at 150 min), suggesting weak or negligible activity. Similarly, the GLT-water phase did not exhibit a significant antinociceptive response, with latency times remaining close to control values throughout the experiment (e.g., 14.08 ± 4.31 s at 60 min and 13.82 ± 2.45 s at 150 min). All results are displayed in Table 4.

Regarding the fraction activities, all results are also shown in Table 4. Only the A3 fraction demonstrated a notable antinociceptive effect, with latency values of 21.90 ± 6.28 s at 60 min and 20.44 ± 5.60 s at 90 min. Conversely, fraction A4 exhibited moderate activity, particularly at 30 min (16.17 ± 6.25 s) and 60 min (18.55 ± 4.72 s), with the latter being statistically significant. Nonetheless, this effect was not sustained throughout the experiment. Fractions A1 and A6 showed slight increases in latency over time, indicating weak and delayed activity. Fraction A2 exhibited variable responses, with moderate increases detected at later time intervals (15.05 ± 5.48 s at 90 min and 15.02 ± 7.01 s at 150 min). In contrast, the A5 fraction remained predominantly inactive throughout the experiment, with latency values closely aligning with those observed in the control group.

The antinociceptive effects of compound A and compound B, assessed using the hot-plate test, are highlighted in Table 5. Compound A (14.55 ± 3.73 s) and compound B (12.17 ± 2.74 s) showed only slight improvements in pain relief compared to the control group (15.47 ± 3.51 s). At 60 min, the antinociceptive effects of the test compounds became more evident. Compound A increased latency time to 18.20 ± 5.42 s, while compound B reached 16.42 ± 3.42 s, both indicating moderate central analgesic activity, although still lower than morphine (25.95 ± 4.13 s). At 90 min, all groups showed increased latency; however, the differences between groups were less notable. Compound A (20.72 ± 6.61 s) and compound B (18.98 ± 6.47 s) remained close to the control (18.52 ± 6.78 s) and morphine (22.74 ± 3.14 s), suggesting a plateau in effect. At 150 min, compound A maintained its activity (21.15 ± 7.32 s), showing a sustained antinociceptive effect comparable to the control (21.02 ± 5.43 s), whereas compound B exhibited a decline (11.70 ± 2.71 s). Morphine also showed a reduction in effect at this time point (18.76 ± 4.88 s).

**Figure 7:**
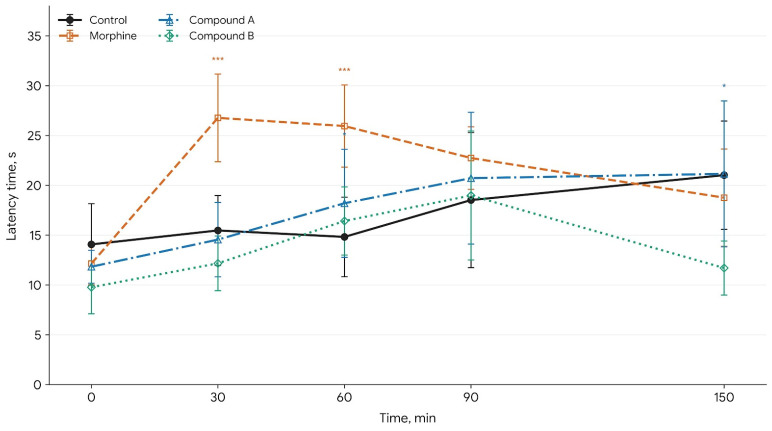
Antinociceptive activity of the isolated compounds in the hot-plate test. Complete datasets for the isolated compounds are provided in Table 5. Values are expressed as mean ± SD (*n* = 6). Significance levels indicate comparison versus control group: * *p* < 0.05, *** *p* < 0.001.

The antinociceptive activities of the isolated compounds in the hot-plate test are graphically presented in Figure 7 to facilitate visualization of their time-dependent effects.

## 3. Discussion

In the present study, the anti-inflammatory activity of *Glaucium* extracts and fractions was evaluated using the carrageenan-induced paw edema model. The anti-inflammatory activity test showed that a dose of 200 mg/kg of GCT was inactive at all measured time points. In contrast, GLT inhibited carrageenan-induced hind paw edema from the second to the sixth hour at the same dose. At 100 mg/kg, both GCT and GLT displayed anti-inflammatory activity only during the first hour, with no effects thereafter. There was no significant difference in paw volume between before GCT 50 mg/kg application and up to 3 h after, indicating that GCT could prevent edema development for up to 3 h at this dose. However, from 4 to 6 h post-application, edema appeared in the right paw, and GCT 50 mg/kg dose treatment no longer alleviated it. GLT at 50 mg/kg showed anti-inflammatory activity only during the first hour after treatment, making its effect at this dose insignificant. The carrageenan-induced paw edema model is one of the most widely used and reliable in vivo methods for evaluating acute inflammatory responses and screening potential anti-inflammatory agents. This model is characterized by a biphasic inflammatory process. The early phase (0–2 h) is predominantly mediated by the release of histamine, serotonin (5-hydroxytryptamine), and bradykinin, whereas the late phase (2–6 h) is mainly associated with enhanced prostaglandin synthesis through activation of the cyclooxygenase (COX) pathway and other arachidonic acid metabolites [12,13,14,15]. The initial inflammatory response is characterized by vascular changes and the development of mild edema, while the subsequent phase is associated with a more pronounced inflammatory reaction resulting from the increased production of pro-inflammatory mediators. Therefore, this model remains a valuable tool for elucidating the mechanisms of anti-inflammatory agents and evaluating their therapeutic potential [14,16]. As expected, the control group treated with carboxymethylcellulose (CMC) exhibited no significant difference between the two paws prior to carrageenan administration, confirming baseline homogeneity. The reference drug, indomethacin, a known nonsteroidal anti-inflammatory drug (NSAID), effectively inhibited edema formation up to the third hour, as evidenced by the absence of significant paw differences during this period. This pattern implies that GLT may interfere with prostaglandin-mediated pathways rather than early inflammatory mediators. The temporary nature of this activity clearly indicates that further fractionation and isolation are essential to identify bioactive compounds.

A similar temporal profile was observed for the GCA 50 mg/kg and GCA 100 mg/kg treatments, both of which delayed edema formation in the early hours but failed to sustain this effect. These findings may indicate the presence of bioactive constituents that target early inflammatory signaling pathways but lack sustained pharmacokinetic or pharmacodynamic properties. In contrast, GLA extracts (both 50 and 100 mg/kg) were consistently ineffective at all time points, indicating that this fraction likely lacks compounds with anti-inflammatory potential, at least within the tested dose range. Taken together, these results highlight a clear dose-dependent and time-dependent variation in anti-inflammatory activity among the tested extracts. The differential activity profiles between GCT, GLT, GCA, and GLA extracts suggest that the distribution of active constituents varies significantly among extracts. In particular, GLT appears to be the most promising candidate for further phytochemical investigation.

At the fraction level, the GLT–EtOAc fraction showed notable anti-inflammatory activity. Sub-fraction analysis revealed that fraction A4 exhibited the strongest effect in this time range, indicating that the active compounds are concentrated in this fraction. In contrast, sub-fractions A1–2 and A5–6 showed weaker activity, suggesting that the main biological effect is largely driven by the constituents present in A4.

The antinociceptive effects of the extracts were evaluated through the utilization of the tail-flick and hot-plate tests. Analyzing the results collectively reveals a time- and dose-dependent analgesic profile, with certain extracts exhibiting differential efficacy between the two assessment models. The tail-flick test demonstrated the most pronounced effects at later time points, specifically at 90 and 150 min. Notably, GLT at 200 mg/kg produced a strong and sustained increase in latency, exceeding that of morphine at the final time point. Similarly, GLA at 50 mg/kg showed a marked late-phase effect. In contrast, lower doses exhibited minimal or transient activity, indicating a clear dose dependency and suggesting that active constituents may require sufficient concentration to exert antinociceptive effects. In the hot-plate test, a broader and generally stronger antinociceptive response was observed across multiple extracts. Among the test samples, GLT 200 again demonstrated prominent activity, particularly at 30 min and 90 min, indicating both early onset and sustained efficacy. Additionally, GCA 50 mg/kg showed a remarkable late-phase effect, surpassing those of most other groups. Compared with the tail-flick results, several extracts, such as GCT 200 mg/kg and GLA 100 mg/kg, showed more consistent, moderate activity in the hot-plate model. When comparing the two models, GLT 200 mg/kg clearly shows the most consistent activity among the extracts. This finding supports the ongoing study focused on the GLT extract.

These assessments provide valuable insights into the mechanisms underlying pain modulation. In the tail-flick test, the GLT-EtOAc fraction showed a significant, time-dependent increase in activity, comparable to morphine at 90 and 150 min. Conversely, the GLT-water and GLT-PE fractions exhibited activity similar to that of the controls, indicating limited or no significant antinociceptive effects. Results from the hot-plate test corroborate that the GLT-EtOAc fraction had the highest activity, although some time points showed no significant differences, indicating possible model-specific variations.

Compounds isolated from the ethyl acetate fraction, identified as kaempferol-3-*O*-glucorhamnoside and quercetin-3-*O*-glucorhamnoside, align with the observed biological effects. In the tail-flick test, kaempferol-3-*O*-glucorhamnoside appeared to have an early onset (30 min) but a short-lived effect, whereas quercetin-3-*O*-glucorhamnoside had a later onset (60 min) with a longer duration. These differences suggest varying pharmacokinetic and pharmacodynamic profiles, possibly due to differences in absorption, distribution, and mechanisms of action. While neither compound significantly outperformed morphine in the tail-flick test, they appear potent as antinociceptive agents. However, their limited impact in the hot-plate test suggests primarily spinal-level effects.

The phytochemical investigation was conducted within the framework of a bioactivity-guided isolation strategy, with the primary aim of identifying constituents associated with the observed biological activities rather than providing an exhaustive characterization of all metabolites present in the extract. Accordingly, the chemical study focused on compounds that were obtained in sufficient quantities to enable complete structural elucidation and subsequent biological evaluation.

The remarkable anti-inflammatory and pain-relieving benefits of other *Glaucium* species have also been reported. *Glaucium grandiflorum* Boiss & Huet, a native Iranian plant, was shown to significantly reduce carrageenan-induced edema, with an ED50 of 13.59 mg/kg. In formalin tests, doses of 60–90 mg/kg of *G. grandiflorum* yielded significant pain relief, and the hot-plate test showed that these doses also significantly elevated pain thresholds [17]. In a study, the pain-relieving effects of the *G. vitellinum* methanol extract were evaluated using the formalin and the hot-plate tests. *G. vitellinum* treatment at 50, 100, 200, and 400 mg/kg by injection provided pain relief during both phases of the formalin test, along with saline and morphine (2 mg/kg by injection) 15 min before the formalin test. In the hot-plate test, *G. vitellinum* extract was tested at doses of 80, 160, 200, and 250 mg/kg. The results showed that all doses of *G. vitellinum* extract significantly reduced pain (*p* < 0.05) during the first phase compared to the control group. In the second phase, the extract’s pain relief was similar to that of morphine. Additionally, using naloxone, a drug that blocks opioid receptors, reversed the pain-relieving effect of the extract in the formalin test. In the hot-plate method, the highest dose of 250 mg/kg showed that the extract’s pain-relieving effect was comparable to morphine. According to the study’s results, *G. vitellinum* extract has significant pain-relieving effects in both the formalin test and the hot-plate method in mice, suggesting it could be a helpful option for pain management [18]. One of the earliest reports demonstrated that extracts obtained from *Glaucium grandiflorum* exhibited significant anti-inflammatory and analgesic activities in experimental animal models. In carrageenan-induced paw edema assays, the plant extract markedly reduced edema formation, indicating inhibition of acute inflammatory responses [17].

Previous pharmacological investigations on *Glaucium* species have largely attributed their anti-inflammatory and analgesic activities to isoquinoline alkaloids, particularly aporphine-, protopine-, and benzophenanthridine-type constituents. Among these, glaucine has received considerable attention for its ability to modulate inflammatory responses by suppressing pro-inflammatory mediators. Ivanovska and Philipov [19] demonstrated that glaucine and oxoglaucine significantly reduced TNF-α and IL-6 production while enhancing IL-10 secretion in activated macrophages, suggesting a regulatory effect on cytokine-mediated inflammatory pathways. Furthermore, comprehensive reviews have highlighted that alkaloids isolated from *Glaucium* species can inhibit nitric oxide production, cyclooxygenase activity, and inflammatory cytokine release, thereby contributing substantially to the anti-inflammatory and analgesic potential of the genus [4,20,21].

Consequently, the biological activities reported for *Glaucium* species have generally been associated with their alkaloid-rich phytochemical profiles. However, the findings of the present study suggest a different perspective. Bioactivity-guided fractionation of *G. leiocarpum* led to the identification of two flavonoid glycosides, kaempferol-3-*O*-glucorhamnoside and quercetin-3-*O*-glucorhamnoside, associated with the observed pharmacological effects. The pharmacological relevance of these findings is supported by prior reports showing that both rutin and kaempferol derivatives exhibit potent anti-inflammatory, antioxidant, and analgesic properties. These compounds have been shown to suppress oxidative stress, inhibit the production of pro-inflammatory cytokines and inflammatory enzymes, and modulate nociceptive signaling pathways.

Quercetin-3-*O*-glucorhamnoside, which is widely known as rutin, is particularly noteworthy due to its well-documented antinociceptive and anti-inflammatory properties. Previous studies have demonstrated that the administration of quercetin-3-*O*-glucorhamnoside produces significant antinociceptive effects, which are partly mediated through opioidergic pathways [22]. In neuropathic pain research, quercetin-3-*O*-glucorhamnoside has demonstrated the ability to reduce mechanical and dynamic allodynia, as well as alleviate spontaneous pain behaviors. This compound also inhibits the activation of mitogen-activated protein kinases and decreases the expression of several pronociceptive mediators within the dorsal root ganglia. Furthermore, administration of the compound has been observed to inhibit upregulation of CX3C chemokine receptor 1, C-C chemokine receptor type 2, and matrix metallopeptidase-9, suggesting a potential modulatory effect on neuroinflammatory signaling pathways implicated in the development of chronic pain [23]. Additional evidence indicates that quercetin-3-*O*-glucorhamnoside may act synergistically with conventional analgesics, as demonstrated by its potentiation of naproxen-induced antinociception in the acetic acid-induced writhing model [24]. More recent studies have also revealed that quercetin-3-*O*-glucorhamnoside treatment significantly reduced paw edema, arthritis scores, thermal hyperalgesia, and cold allodynia, producing effects comparable to those of indomethacin. These improvements were accompanied by a marked reduction in serum TNF-α levels and oxidative stress markers, including malondialdehyde, together with increased superoxide dismutase and catalase activities and restoration of thiol levels. Such findings suggest that quercetin-3-*O*-glucorhamnoside exerts its pharmacological effects through a multifactorial mechanism involving the suppression of inflammatory mediators, attenuation of oxidative stress, and modulation of nociceptive pathways [25].

The other compound, whose structure has been elucidated as kaempferol-3-*O*-rutinoside, namely nicotiflorin, has attracted increasing attention because of its multifaceted anti-inflammatory and antinociceptive properties. Early pharmacological investigations demonstrated that kaempferol-3-*O*-rutinoside-containing extracts significantly reduced nociceptive responses in chemical and thermal pain models, including acetic acid-induced writhing, formalin, capsaicin, and hot-plate tests, suggesting the involvement of both peripheral and central analgesic mechanisms as well as indicating that kaempferol-3-*O*-rutinoside may interfere with the release of inflammatory mediators and the activation of sensory neurons associated with pain transmission [26]. Subsequently, Yu et al. [27] reported that kaempferol-3-*O*-rutinoside protected vascular endothelial cells against TNF-α-induced injury by reducing nitric oxide (NO) production and preserving endothelial integrity, thereby highlighting its role in regulating inflammation-associated vascular dysfunction. Further evidence demonstrated that kaempferol-3-*O*-rutinoside exerts anti-inflammatory effects through the suppression of key inflammatory mediators, including cyclooxygenase-2 (COX-2), inducible nitric oxide synthase (iNOS), TNF-*α*, IL-1*β*, and IL-6, thereby attenuating inflammatory signaling and oxidative stress [28,29]. More recently, mechanistic studies have revealed that kaempferol-3-*O*-rutinoside directly targets major inflammatory pathways, particularly the NF-κB and NLRP3 inflammasome signaling cascades. In an experimental model of ulcerative colitis, kaempferol-3-*O*-rutinoside significantly alleviated tissue inflammation by inhibiting NF-κB activation and downstream inflammasome-mediated responses [30]. Collectively, these studies indicate that kaempferol-3-*O*-rutinoside acts through multiple complementary mechanisms involving cytokine regulation, suppression of inflammatory enzymes, inhibition of oxidative stress, and modulation of nociceptive pathways.

Although members of the Papaveraceae are recognized for their characteristic alkaloids, the present study adopted a bioactivity-guided isolation strategy. While an alkaloid fraction was included in the initial pharmacological screening, the ethyl acetate fraction exhibited the most promising biological activity and was therefore selected for further phytochemical investigation. Consequently, the compounds reported herein reflect the outcome of the bioactivity-guided isolation process rather than a comprehensive characterization of all metabolite classes present in the plant. Within this context, the flavonoid glycosides isolated from the ethyl acetate fraction provide further evidence that, in addition to alkaloids, non-alkaloidal constituents may also contribute to the pharmacological properties of *G. leiocarpum*. To further support the phytochemical characterization, HPLC chromatographic fingerprints of the crude extract and the principal fractions have been included in the Supplementary Materials. These chromatographic data provide additional information on the overall chemical profiles of the investigated samples. Taken together, while alkaloids remain important chemotaxonomic markers of the genus, the present study provides evidence that flavonoid glycosides can also play a meaningful role in mediating the anti-inflammatory and antinociceptive effects of *G. leiocarpum*. Given that quercetin-3-*O*-rutinoside and kaempferol-3-*O*-rutinoside were identified as active constituents of *G. leiocarpum* in the present study, the previously reported mechanisms, as mentioned, may partly explain the antinociceptive and anti-inflammatory activities observed in the current experimental models. Therefore, the biological activity of *G. leiocarpum* may not be exclusively attributable to alkaloidal constituents, as traditionally assumed, but may also involve substantial contributions from flavonoid-derived mechanisms. This observation expands the current understanding of the pharmacologically active constituents of the genus and suggests that non-alkaloidal metabolites should also be considered when investigating the therapeutic potential of *Glaucium* species. Further studies focusing on isolated compounds, synergistic interactions between flavonoids and alkaloids, and the molecular mechanisms of action are warranted to clarify their relative contributions to the plant’s overall biological activity.

Although the primary objective of the present study was to identify the bioactive extracts, fractions, and compounds responsible for the observed anti-inflammatory and antinociceptive activities through a bioactivity-guided isolation approach, a comprehensive toxicological evaluation was beyond the scope of this work. The selection of fractions for further investigation was based on their pharmacological activity, and the alkaloid fraction was not selected for subsequent phytochemical investigation because it was not identified as the most active fraction during the bioactivity-guided screening. Furthermore, no mortality or obvious signs of acute toxicity were observed in the experimental animals at the tested doses. Likewise, although complementary in vitro anti-inflammatory assays, such as HRBC membrane stabilization and LPS-stimulated macrophage models, may provide additional mechanistic insights while reducing animal use, the present study was designed to evaluate the pharmacological efficacy of the extracts, fractions, and isolated compounds using well-established in vivo models. The carrageenan-induced paw edema model enables the assessment of the complex inflammatory response under physiological conditions, while the tail-flick and hot-plate tests allow simultaneous evaluation of antinociceptive activity. Therefore, these in vivo models were considered the most appropriate approach to achieve the objectives of the study. Nevertheless, future investigations should include complementary in vitro studies to further elucidate the underlying mechanisms of action, as well as comprehensive toxicological evaluations to establish the safety profiles of the active fractions and isolated compounds.

In conclusion, the findings of this study suggest that *G. leiocarpum*, particularly its total extract, exhibits promising antinociceptive activity with a dose- and time-dependent profile. Although the effects were not statistically confirmed at the pairwise level, the observed trends warrant further investigation, including isolation of active constituents and evaluation of their mechanisms of action.

## 4. Materials and Methods

### 4.1. Plant Material

*Glaucium* species were collected from the Yaşamkent district of Ankara, Türkiye, during the flowering period between May and June 2020, following the required permissions from the Ministry of Agriculture and Forestry. The collected plant materials were taxonomically identified by Prof. Dr. Hayri Duman (Department of Biology, Faculty of Science, Gazi University, Ankara, Türkiye). Voucher specimens of *Glaucium corniculatum* (L.) Rudolph subsp. *refractum* (NAB) Cullen (AEF 28819) and *Glaucium leiocarpum* Boiss. (AEF 28817) are deposited in the Herbarium of Ankara University Faculty of Pharmacy (AEF), Ankara, Türkiye.

### 4.2. Experimental Design

The study was designed as a controlled in vivo experimental investigation to evaluate the anti-inflammatory and antinociceptive activities of the plant extracts, alkaloid fractions, solvent fractions, chromatographic subfractions, and isolated compounds. The independent variables were treatment type, administered dose, and measurement time. The dependent variables were paw volume in the carrageenan-induced paw edema model and response latency in the tail-flick and hot-plate tests. The vehicle-treated group served as the negative control, while indomethacin and morphine were used as reference treatments for the anti-inflammatory and antinociceptive experiments, respectively. Each experimental group consisted of six animals. Measurements were obtained repeatedly at predefined time points, and the results were expressed as mean ± SD.

All animal experiments were conducted following the approval of the Ankara University Local Ethics Committee for Animal Experiments (Approval No. 2020-1-3) and in accordance with the principles of the 3Rs (Replacement, Reduction, and Refinement). Although no validated in vitro model could replace the integrated evaluation of both anti-inflammatory and antinociceptive activities required for the objectives of this study, efforts were made to minimize animal use and suffering. To comply with the principle of Reduction, the tail-flick and hot-plate tests were performed sequentially in the same animals following treatment administration, thereby reducing the total number of animals required. In addition, the minimum number of animals necessary to achieve statistically reliable results (n = 6 per group) was used, and all procedures were conducted to minimize animal distress in accordance with ethical guidelines.

### 4.3. Extraction and Isolation

**Figure 8:**
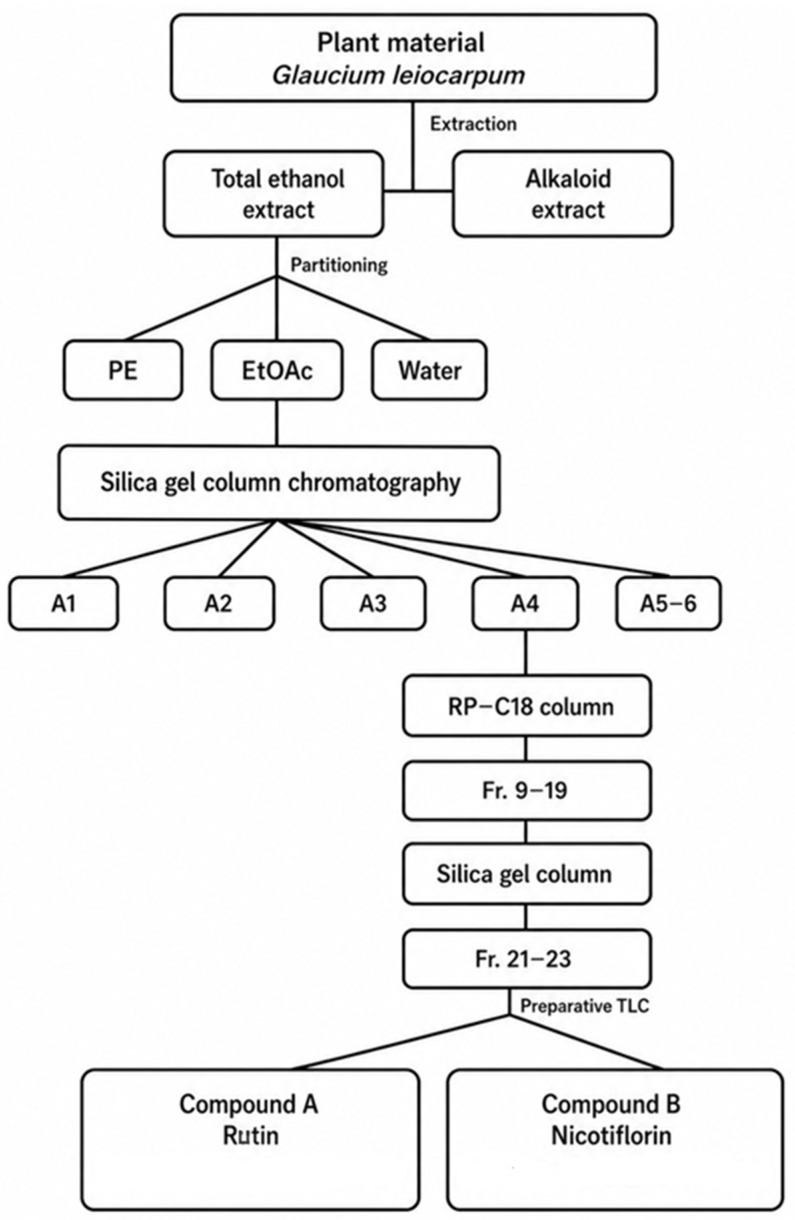
Schematic representation of the bioactivity-guided extraction, fractionation, and isolation procedure of *Glaucium leiocarpum*, leading to the isolation of rutin (compound A) and nicotiflorin (compound B).

The overall bioactivity-guided extraction and isolation workflow is summarized in Figure 8, while the individual steps are described in detail in the following subsections.

#### 4.3.1. Preparation of Total Ethanolic Extract

After drying in the shade at room temperature, the aerial parts of both *Glaucium* species, including the petals, were mechanically powdered. Approximately 1 kg of powdered plant material was extracted with 95% ethanol using a Soxhlet apparatus over three consecutive 8 h cycles. The combined ethanolic extracts were concentrated under reduced pressure at 45 °C using a rotary evaporator to obtain crude total ethanolic extracts. Total extracts were coded as GCT for *G. corniculatum* and GLT for *G. leiocarpum*.

#### 4.3.2. Preparation of Total Alkaloid Fraction

Crude alkaloid fractions were prepared from the total ethanolic extracts. Briefly, the concentrated ethanolic extract was acidified with 3% HCl solution to pH 1–2 and kept at room temperature overnight. The mixture was then filtered, and the filtrate was subjected to liquid–liquid extraction with hexane to remove nonpolar constituents. Subsequently, the aqueous phase was alkalized to pH 9–10 using 25% NH_4_OH and extracted repeatedly with dichloromethane. The dichloromethane phases were combined and evaporated to dryness under reduced pressure, yielding crude alkaloid fractions. Total alkaloid fractions were coded as GCA for *G. corniculatum* and GLA for *G. leiocarpum*.

#### 4.3.3. Fractionation of Total Ethanolic Extract

Due to its prominent biological activity, the total ethanolic extract of *G. leiocarpum* was selected for bioactivity-guided fractionation. The crude extract (GLT; 134.5 g) was suspended in distilled water and successively partitioned with petroleum ether and ethyl acetate. The petroleum ether extraction was repeated until the organic phase became colorless. Combined petroleum ether phases were concentrated under reduced pressure at 40 °C. The remaining aqueous phase was subsequently partitioned with ethyl acetate using the same procedure. Finally, the residual aqueous phase was lyophilized. As a result, petroleum ether (PE), ethyl acetate (EtOAc), and aqueous fractions were obtained.

#### 4.3.4. Isolation of Active Compounds from EtOAc Fraction of Total Ethanolic Extract

The ethyl acetate fraction (GLT-EtOAc; 21.75 g), which exhibited the highest activity, was subjected to chromatographic isolation procedures. Initially, the fraction was separated on a silica gel column using a CHCl_3_–MeOH gradient system (100:0 → 0:100, *v*/*v*). Fractions with similar TLC profiles were combined to obtain six major subfractions (A1–A6). The subfractions were evaluated biologically, and subfraction GL-A4 (51.80 g) was selected for further purification due to its remarkable activity. Fraction monitoring was performed by thin-layer chromatography (TLC) using EtOAc–formic acid–acetic acid–water (100:11:11:26, *v*/*v*/*v*/*v*) as the mobile phase. TLC plates were visualized under UV light at 254 and 366 nm and sprayed with vanillin–H_2_SO_4_ reagent.

Subfraction A4 (fractions 35–37) was further purified by reversed-phase column chromatography on C18 silica gel using an H_2_O–MeOH gradient system (90:10 → 0:100, *v*/*v*). Subfractions were collected according to their chromatographic behavior and subsequently combined.

Further purification was achieved by column chromatography on silica gel, eluted with CHCl_3_–MeOH–H_2_O (80:20:2 → 70:30:5, *v*/*v*/*v*), using the combined fraction Fr. 9–19 (1.19 g). Final purification of the compounds was performed by preparative thin-layer chromatography using EtOAc–MeOH–water (100:15:10, *v*/*v*/*v*) as the developing solvent system.

As a result of the isolation studies, two flavonoid derivatives were isolated and structurally characterized by spectroscopic methods, including ^1^H-NMR, ^13^C-NMR, 2D-NMR (COSY, HSQC, HMBC), and LC-MS analyses.

### 4.4. Biological Activity Studies

#### 4.4.1. Anti-Inflammatory Activity

##### Carrageenan-Induced Paw Edema Model

Male Wistar albino rats were used to evaluate anti-inflammatory activity. Animals were randomly divided into 12 groups (*n* = 6): control, reference, total ethanolic extracts (50, 100, and 200 mg/kg), and alkaloid fractions (50 and 100 mg/kg). Samples were suspended in physiological saline containing carboxymethyl cellulose (CMC) and administered intraperitoneally (i.p.) 1 h before carrageenan injection. Indomethacin (10 mg/kg, i.p.) was used as the reference drug.

Acute inflammation was induced by subcutaneous injection of 0.05 mL of 1% carrageenan solution (Sigma, Burbank, CA, USA) into the plantar surface of the right hind paw. The same volume of saline solution was injected into the left hind paw as a control. Paw edema volume was measured hourly for the first 6 h using a plethysmometer (Ugo Basile, Gemonio-Lombardy, Italy).

#### 4.4.2. Antinociceptive Activity

##### Tail-Flick Test

Male Balb/c mice were divided into 12 groups (*n* = 6). Test samples, including total extracts (50, 100, and 200 mg/kg), alkaloid fractions (50 and 100 mg/kg), and isolated compounds (10 mg/kg), were suspended in CMC-containing saline and administered intraperitoneally. Morphine hydrochloride (10 mg/kg, s.c.) was used as the reference drug.

Reaction times were recorded at 0 min (before administration) and at 30, 60, 90, and 150 min after treatment. During the experiment, radiant heat was applied to a marked 5 cm region of the tail using a tail-flick analgesia meter (Ugo Basile, Gemonio, Italy). The latency period between heat application and tail withdrawal was recorded as the reaction time. The infrared intensity was set at IR = 15 based on preliminary optimization studies. To avoid tissue damage, a 30 s cut-off time was used.

##### Hot-Plate Test

Male Balb/c mice were divided into experimental groups as described above. Test samples and reference drugs were administered using the same protocol as in the tail-flick assay. Animals were placed individually on a hot plate maintained at 50 °C, and response latencies to thermal stimuli (paw licking or jumping) were recorded at 0, 30, 60, 90, and 150 min after treatment. A maximum cut-off time of 30 s was applied to prevent tissue injury.

### 4.5. Statistical Analysis

Statistical analyses were performed using SPSS version 21.0 software (IBM Corp., Armonk, NY, USA). Data are presented as mean ± standard deviation (SD) and standard error of the mean (SEM).

The normality of the data obtained from anti-inflammatory activity studies was evaluated using the Shapiro–Wilk test. Comparisons between right and left paw volumes within the same group were performed using Student’s *t*-test.

Repeated-measures ANOVA and one-way ANOVA were used for intergroup comparisons. Homogeneity of variances was assessed by Levene’s test. Tukey’s HSD post hoc test was used to make multiple comparisons between groups. Differences were considered statistically significant at *p* < 0.05.

## Figures and Tables

**Table 1:** Anti-inflammatory activity of the total ethanolic extracts and alkaloid extracts of *G. leiocarpum* (GLT), *G. corniculatum* (GCT), subextracts of GLT and fractions of GLT-EtOAc (comparison of test and control paw volume measurements at different time points).

Group	0 h	1 h	2 h	3 h	4 h	5 h	6 h
Control (CMC)	1.67 ± 0.16	1.87 ± 0.22 *	2.04 ± 0.17 *	2.24 ± 0.37 *	2.38 ± 0.49 *	2.42 ± 0.54 *	2.46 ± 0.51 *
Indomethacin	1.38 ± 0.12	1.54 ± 0.14	1.58 ± 0.14	1.67 ± 0.19 *	1.68 ± 0.12 *	1.75 ± 0.10 *	1.89 ± 0.10 *
GCT 200	1.65 ± 0.07	1.72 ± 0.13 *	1.80 ± 0.12 *	1.81 ± 0.20 *	1.86 ± 0.15 *	1.92 ± 0.27 *	1.95 ± 0.29 *
GLT 200	1.54 ± 0.32	1.58 ± 0.30 *	1.56 ± 0.33	1.71 ± 0.38	1.76 ± 0.34	1.75 ± 0.39	1.73 ± 0.51
GCT 100	1.53 ± 0.05	1.67 ± 0.09	1.74 ± 0.07 *	1.80 ± 0.08 *	1.90 ± 0.13 *	1.97 ± 0.11 *	2.04 ± 0.13 *
GLT 100	1.51 ± 0.02	1.68 ± 0.11	1.75 ± 0.10 *	1.79 ± 0.12 *	1.86 ± 0.21 *	1.94 ± 0.25 *	1.96 ± 0.24 *
GCT 50	1.57 ± 0.10	1.74 ± 0.14	1.50 ± 0.75	2.01 ± 0.22 *	2.13 ± 0.30 *	2.27 ± 0.25 *	2.41 ± 0.30 *
GLT 50	1.43 ± 0.10	1.55 ± 0.09	1.65 ± 0.12 *	1.81 ± 0.18 *	1.86 ± 0.21 *	2.04 ± 0.21 *	2.07 ± 0.24 *
GCA 100	1.75 ± 0.19	1.95 ± 0.21	2.01 ± 0.22 *	2.04 ± 0.24	2.11 ± 0.18 *	2.20 ± 0.25 *	2.24 ± 0.22 *
GLA 100	1.57 ± 0.09	1.70 ± 0.09 *	1.78 ± 0.19 *	1.85 ± 0.19 *	1.95 ± 0.20 *	2.00 ± 0.16 *	2.07 ± 0.18 *
GCA 50	1.69 ± 0.06	1.81 ± 0.07	1.98 ± 0.18	2.05 ± 0.24	2.18 ± 0.21 *	2.20 ± 0.15 *	2.27 ± 0.10 *
GLA 50	1.58 ± 0.14	1.68 ± 0.15 *	1.72 ± 0.12 *	1.79 ± 0.15 *	1.93 ± 0.12 *	1.97 ± 0.09 *	2.12 ± 0.08 *
PE	1.69 ± 0.19	1.82 ± 0.24	1.82 ± 0.17	1.83 ± 0.17	1.88 ± 0.18	1.86 ± 0.22	1.92 ± 0.24
ETOAc	1.46 ± 0.18	1.53 ± 0.16 *	1.59 ± 0.12	1.59 ± 0.10	1.59 ± 0.11	1.60 ± 0.10 *	1.65 ± 0.11
Water	1.53 ± 0.21	1.63 ± 0.21	1.68 ± 0.21 *	1.72 ± 0.20	1.73 ± 0.24	1.76 ± 0.28	1.89 ± 0.35 *
A1-A2	1.59 ± 0.08	1.71 ± 0.08 **	1.89 ± 0.22 *	1.96 ± 0.14 ***	2.05 ± 0.15 ***	2.13 ± 0.14 ***	2.14 ± 0.16 ***
A3	1.50 ± 0.07	1.52 ± 0.04	1.53 ± 0.05	1.55 ± 0.05 *	1.58 ± 0.07 *	1.64 ± 0.09 *	1.64 ± 0.12 **
A4	1.45 ± 0.05	1.62 ± 0.21 *	1.61 ± 0.25	1.63 ± 0.29	1.64 ± 0.32	1.63 ± 0.32	1.71 ± 0.34
A5-A6	1.59 ± 0.12	1.65 ± 0.14	1.77 ± 0.16 **	1.77 ± 0.21	1.92 ± 0.24 **	1.98 ± 0.25 **	2.01 ± 0.24 **

Values are expressed as mean ± SD (*n* = 6). Statistical comparisons were performed between right and left paws (paired analysis). * *p* < 0.05; ** *p* < 0.01, *** *p* < 0.001: statistically significant difference, no symbol: not significant (*p* > 0.05).

**Table 2:** Tail-flick test results of total ethanolic extracts and alkaloid extracts of *G. leiocarpum* (GLT), *G. corniculatum* (GCT), subextracts of GLT and fractions of GLT-EtOAc.

	Time	0th min	30th min	60th min	90th min	150th min
Group						
Control (CMC)		11.97 ± 2.95	10.19 ± 2.33	11.28 ± 2.15	12.36 ± 3.60	10.05 ± 1.36
Morphine		10.05 ± 2.08	15.99 ± 7.62	20.13 ± 5.12	17.91 ± 4.37	12.20 ± 2.18 *
GLT 200		12.73 ± 2.62	20.73 ± 5.94	20.25 ± 7.78	23.91 ± 5.09	22.23 ± 4.84
GCT 200		13.06 ± 7.90	19.90 ± 6.31	19.06 ± 6.42	16.18 ± 8.13	18.85 ± 7.87
GLT 100		10.08 ± 3.89	14.43 ± 8.03	14.16 ± 5.88	14.70 ± 4.63	15.30 ± 5.87
GCT 100		12.40 ± 1.12	15.18 ± 3.89	15.11 ± 4.52	15.21 ± 3.88	15.18 ± 3.48
GLT 50		14.83 ± 4.46	15.91 ± 3.43	14.36 ± 2.73	11.58 ± 2.26	14.90 ± 2.51
GCT 50		11.91 ± 4.10	15.00 ± 1.43	15.35 ± 2.89	13.13 ± 3.42	11.53 ± 1.71
GLA 100		10.90 ± 2.21	14.40 ± 3.32	17.33 ± 4.39	15.98 ± 3.48	16.70 ± 5.43
GCA 100		14.06 ± 2.39	17.90 ± 6.38	17.75 ± 5.88	16.76 ± 2.66	15.55 ± 1.78
GLA 50		10.93 ± 3.38	13.53 ± 5.61	16.03 ± 4.80	15.96 ± 8.83	21.26 ± 7.51
GCA 50		12.25 ± 0.85	10.98 ± 2.21	13.11 ± 3.95	15.03 ±1.53	15.70 ±2.01
GLT-PE		7.97 ± 1.16	11.60 ± 2.82 *	12.28 ± 2.56 **	11.70 ± 3.29 *	11.28 ± 1.44 **
GLT-EtOAc		9.97 ± 3.00	12.30 ± 3.93 *	17.57 ± 4.70 **	20.28 ± 6.74 **	21.00 ± 7.80 *
GLT-Water		9.85 ± 1.91	13.15 ± 2.56 *	11.43 ± 3.61	10.85 ± 2.43	10.27 ± 1.16
A1		11.33 ± 2.35	11.03 ± 1.84	12.60 ± 2.53	14.30 ± 2.30	14.92 ± 2.67
A2		8.12 ± 1.34	10.02 ± 2.92	12.27 ± 2.77	11.85 ± 1.81	12.70 ± 1.61
A3		10.48 ± 1.47	11.75 ± 1.57	13.79 ± 2.80	14.83 ± 1.55	13.38 ± 2.81
A4		10.75 ± 2.53	18.88 ± 6.09 **	14.75 ± 7.73 *	15.33 ± 3.10 **	14.92 ± 3.99
A5		11.82 ± 2.44	11.73 ± 0.70	16.10 ± 6.97 *	14.41 ± 4.00 **	15.12 ± 9.46
A6		10.13 ± 1.03	13.65 ± 2.49	12.83 ± 2.02	10.83 ± 1.87	9.91 ± 2.17

Values are expressed as mean ± SD (*n* = 6). Statistical analysis was performed using one-way ANOVA followed by appropriate post hoc test. Significance levels indicate comparison versus control group: * *p* < 0.05, ** *p* < 0.01 versus baseline (0 min) within the same group (Bonferroni post hoc test). Latency times are expressed in seconds (s).

**Table 3:** Tail-flick test results of isolated compounds.

Group	0 min	30 min	60 min	90 min	150 min
Control (CMC)	11.98 ± 2.95	10.19 ± 2.33	11.28 ± 2.15	12.37 ± 3.60	10.05 ± 1.37
Morphine	10.05 ± 2.08	15.99 ± 7.62 *	20.13 ± 5.12 **	17.92 ± 4.38 ***	12.20 ± 2.19 *
Compound A	9.88 ± 1.27	13.87 ± 2.21	23.17 ± 5.97 **	26.25 ± 4.93 ***	24.10 ± 9.09 ***
Compound B	11.80 ± 1.56	19.12 ± 5.46 *	22.90 ± 7.46 **	25.68 ± 6.98 ***	17.12 ± 8.00 **

Values are expressed as mean ± SD (*n* = 6). Statistical analysis was performed using one-way ANOVA followed by appropriate post hoc test. Significance levels indicate comparison versus control group: * *p* < 0.05, ** *p* < 0.01, *** *p* < 0.001 versus baseline (0 min) within the same group (Bonferroni post hoc test). Latency times are expressed in seconds (s).

**Table 4:** Hot-plate test results of total ethanolic extracts and alkaloid extracts of GLT, GCT, subextracts of GLT and fractions of GLT-EtOAc.

Group	0 min	30 min	60 min	90 min	150 min
Control (CMC)	14.07 ± 4.09	15.47 ± 3.51	14.82 ± 3.99	18.52 ± 6.78	21.02 ± 5.43
Morphine	12.14 ± 2.10	26.77 ± 4.40 ^a^	25.95 ± 4.13 ^a^	22.74 ± 3.14 ^a^	18.76 ± 4.88 ^c^
GLT 200	14.00 ± 3.30	22.27 ± 4.76 ^a^	19.53 ± 2.64	23.00 ± 4.61 ^a^	14.21 ± 2.02 ^d^
GCT 200	15.12 ± 3.27	19.77 ± 4.35	20.27 ± 6.05	20.55 ± 4.75	20.23 ± 5.06
GLT 100	13.13 ± 3.92	16.53 ± 5.52	18.53 ± 5.58	20.47 ± 5.42	16.83 ± 8.68
GCT 100	19.48 ± 6.89	16.23 ± 3.29	19.57 ± 10.20	16.98 ± 3.31	16.07 ± 4.69
GLT 50	10.67 ± 3.96	9.87 ± 1.55	11.03 ± 1.73	10.02 ± 3.79	11.26 ± 3.15
GCT 50	9.58 ± 0.44	9.33 ± 1.57	8.50 ± 1.63	9.47 ± 3.00	10.66 ± 2.91
GLA 100	14.63 ± 3.99	18.55 ± 6.00	22.95 ± 7.09	19.02 ± 3.83	19.67 ± 7.26
GCA 100	13.08 ± 2.57	15.90 ± 4.08	19.33 ± 4.25 ^a^	14.77 ± 4.00	13.17 ± 3.03
GLA 50	8.90 ± 2.33	12.37 ± 1.42 ^a^	17.27 ± 5.76	19.38 ± 3.73 ^ab^	19.58 ± 5.34 ^a^
GCA 50	12.78 ± 3.30	17.08 ± 1.10	19.06 ± 6.68	20.32 ± 6.00	22.99 ± 2.08
GLT-PE	9.62 ± 1.81	12.63 ± 2.46	13.97 ± 2.23	13.95 ± 3.93	11.03 ± 3.37
GLT-EtOAc	10.97 ± 1.98	13.98 ± 2.80	19.58 ± 4.14 *	17.15 ± 1.90	20.47 ± 7.25 **
GLT-Water	12.95 ± 2.30	15.03 ± 3.38	14.08 ± 4.31	15.90 ± 2.40	13.82 ± 2.45
A1	11.03 ± 1.41	11.12 ± 1.57	13.87 ± 3.70	14.53 ± 2.62	16.42 ± 3.46
A2	9.80 ± 2.73	9.78 ± 0.70	12.65 ± 6.44	15.05 ± 5.48	15.02 ± 7.01
A3	11.95 ± 2.28	15.07 ± 1.88	21.90 ± 6.28 **	20.44 ± 5.60 **	18.18 ± 5.24
A4	10.85 ± 2.13	16.17 ± 6.25	18.55 ± 4.72 *	14.03 ± 3.13	15.27 ± 3.62
A5	10.23 ± 2.32	11.49 ± 3.04	11.35 ± 2.80	11.12 ± 3.12	12.27 ± 2.12
A6	10.67 ± 1.30	14.39 ± 3.11	14.05 ± 4.15	13.83 ± 4.19	11.43 ± 2.93

Data are expressed as mean ± SD (*n* = 6). ^a^ *p* < 0.05 versus 0 min. ^b^ *p* < 0.05 versus 30 min. ^c^ *p* < 0.05 versus 60 min. ^d^ *p* < 0.05 versus 90 min. (Bonferroni post hoc test). Values are expressed as mean ± SD (*n* = 6), Latency times are given in seconds (s). Significance vs. control group: * *p* < 0.05; ** *p* < 0.01.

**Table 5:** Hot-plate test results of isolated compounds.

Group	0 min	30 min	60 min	90 min	150 min
Control (CMC)	14.07 ± 4.09	15.47 ± 3.51	14.82 ± 3.99	18.52 ± 6.78	21.02 ± 5.43
Morphine	12.14 ± 2.10	26.77 ± 4.40 ***	25.95 ± 4.13 ***	22.74 ± 3.14	18.76 ± 4.88
Compound A	11.83 ± 1.65	14.55 ± 3.73	18.20 ± 5.42 *	20.72 ± 6.61	21.15 ± 7.32 *
Compound B	9.77 ± 2.66	12.17 ± 2.74	16.42 ± 3.42	18.98 ± 6.47	11.70 ± 2.71

Values are expressed as mean ± SD (*n* = 6). Significance levels indicate comparison versus. control group: * *p* < 0.05, *** *p* < 0.001.

## Data Availability

The data presented in this study are available from the corresponding author upon reasonable request.

## Supplementary Materials

The following supporting information can be downloaded at https://www.mdpi.com/article/10.3390/molecules31152711/s1: Figure S1. HPLC chromatogram (254 nm) of the total ethanolic extract of *Glaucium leiocarpum*; Figure S2. HPLC chromatogram (254 nm) of the ethyl acetate subextract of *Glaucium leiocarpum*; Figure S3. HPLC chromatogram (254 nm) of fraction A4 obtained from the ethyl acetate subextract of *Glaucium leiocarpum*; Figure S4. HPLC chromatogram (254 nm) of fraction Fr9–19 obtained from fraction A4 of the ethyl acetate subextract of *Glaucium leiocarpum*; Figure S5. HPLC chromatogram (254 nm) and UV spectrum of rutin (Compound A); Figure S6. HPLC chromatogram (254 nm) and UV spectrum of nicotiflorin (Compound B); Figure S7. ^1^H NMR spectrum of rutin (Compound A); Figure S8. ^13^C NMR spectrum of rutin (Compound A); Figure S9. ^1^H NMR spectrum of nicotiflorin (Compound B); Figure S10. ^13^C NMR spectrum of nicotiflorin (Compound B).

## References

[1] Yi T., Zhao Z.Z., Yu Z.L., Chen H.B. (2010). Comparison of the Anti-Inflammatory and Anti-Nociceptive Effects of Three Medicinal Plants Known as “Snow Lotus” Herb in Traditional Uighur and Tibetan Medicines. J. Ethnopharmacol..

[2] Mehrara M., Halakoo M., Hakemi-Vala M., Hashemi S.J., Asgarpanah J. (2015). Antibacterial and Antifungal Activities of the Endemic Species *Glaucium vitellinum* Boiss. and Buhse. Avicenna J. Phytomed..

[3] Elbermawi A., Elsbaey M., Moussa M., Tammam M., Assaf H.K. (2018). Phytochemical and Biological Studies on *Glaucium* Species: A Review. Nat. Prod. Res..

[4] Akaberi T., Shourgashti K., Emami S.A., Akaberi M. (2021). Phytochemistry and Pharmacology of Alkaloids from *Glaucium* spp.. Phytochemistry.

[5] Tuzlacı E., Doğan A. (2010). Turkish Folk Medicinal Plants, IX: Ovacık (Tunceli). Marmara Pharm. J..

[6] Altundağ E., Öztürk M. (2011). Ethnomedicinal Studies on the Plant Resources of East Anatolia, Turkey. Procedia Soc. Behav. Sci..

[7] Sargın S.A. (2015). Ethnobotanical Survey of Medicinal Plants in Bozyazı District of Mersin, Turkey. J. Ethnopharmacol..

[8] Bulut G., Haznedaroğlu M.Z., Doğan A., Koyu H., Tuzlacı E. (2017). An Ethnobotanical Study of Medicinal Plants in Acipayam (Denizli, Turkey). J. Herb. Med..

[9] Erbay M.Ş., Anıl S., Melikoğlu G. (2018). Plants Used as Painkiller in Traditional Treatment in Turkey-II: Headache. Marmara Pharm. J..

[10] Said O., Khalil K., Fulder S., Azaizeh H. (2002). Ethnopharmacological Survey of Medicinal Herbs in Israel, the Golan Heights and the West Bank Region. J. Ethnopharmacol..

[11] Hayta S., Polat R., Selvi S. (2014). Traditional Uses of Medicinal Plants in Elazığ (Turkey). J. Ethnopharmacol..

[12] Posadas I., Bucci M., Roviezzo F., Rossi A., Parente L., Sautebin L., Cirino G. (2004). Carrageenan-Induced Mouse Paw Oedema is Biphasic, Age-Weight Dependent and Displays Differential Nitric Oxide and Cyclooxygenase-2 Expression. Br. J. Pharmacol..

[13] Hafeez A., Jain U., Sajwan P., Srivastava S., Thakur A. (2013). Evaluation of Carrageenan-Induced Anti-Inflammatory Activity of Ethanolic Extract of Bark of Ficus virens Linn. in Swiss Albino Mice. J. Phytopharm..

[14] Hanh C.H., Khaziakhmetova V.N., Ziganshina L.E. (2015). Modeling Inflammatory Edema: Are the Models Interchangeable?. Eksp. Klin. Farmakol..

[15] Patil K.R., Mahajan U.B., Unger B.S., Goyal S.N., Belemkar S., Surana S.J., Ojha S., Patil C.R. (2019). Animal Models of Inflammation for Screening of Anti-Inflammatory Drugs: Implications for the Discovery and Development of Phytopharmaceuticals. Int. J. Mol. Sci..

[16] Özbek H., Öztürk A. (2003). Antienflamatuvar Etkinliğin Ölçülmesinde Kullanılan Yöntemler. Van. Tıp Derg..

[17] Morteza-Semnani K., Saeedi M., Hamidian M., Vafamehr H., Dehpour A.R. (2002). Anti-Inflammatory, Analgesic Activity and Acute Toxicity of *Glaucium grandiflorum* Extract. J. Ethnopharmacol..

[18] Rangriz E., Mousavi Z., Najafizadeh P., Asgarpanah J. (2016). Antinociceptive Effect of the Endemic Species *Glaucium vitellinum* Boiss. and Buhse. Jundishapur J. Nat. Pharm. Prod..

[19] Ivanovska N., Philipov S. (2009). Toll-Like Receptor-Mediated Anti-Inflammatory Action of Glaucine and Oxoglaucine. Fitoterapia.

[20] Souto A.L., Tavares J.F., da Silva M.S., Diniz M.F.F.M., de Athayde-Filho P.F., Barbosa-Filho J.M. (2011). Anti-Inflammatory Activity of Alkaloids: An Update from 2000 to 2010. Molecules.

[21] Orhan I.E., Senol F.S., Ozturk N., Celik S.A., Pulur A., Kan Y., Altun M.L. (2018). Antioxidant, Anti-Inflammatory and Enzyme Inhibitory Activities of *Glaucium grandiflorum* var. *grandiflorum*. Iran. J. Pharm. Res..

[22] Hernandez-Leon A., Fernandez-Guasti A., Gonzalez-Trujano M.E. (2016). Rutin Antinociception Involves Opioidergic Mechanism and Descending Modulation of Ventrolateral Periaqueductal Grey Matter in Rats. Eur. J. Pain..

[23] Lim E.Y., Lee C., Kim Y.T. (2022). The Antinociceptive Potential of Camellia japonica Leaf Extract, (−)-Epicatechin, and Rutin against Chronic Constriction Injury-Induced Neuropathic Pain in Rats. Antioxidants.

[24] Alonso-Castro A.J., Rangel-Velazquez J.E., Isiordia-Espinoza M.A., Villanueva-Solis L.E., Aragon-Martinez O.H., Zapata-Morales J.R. (2017). Synergism between Naproxen and Rutin in a Mouse Model of Visceral Pain. Drug Dev. Res..

[25] Forouzanfar F., Pourbagher-Shahri A.M., Ahmadzadeh A.M. (2025). Rutin attenuates complete Freund’s adjuvant-induced inflammatory pain in rats. Iran. J. Basic. Med. Sci..

[26] do Nascimento J.E.T., de Morais S.M., de Lisboa D.S., de Oliveira Sousa M., Santos S.A.A.R., Magalhães F.E.A., Campos A.R. (2018). The orofacial antinociceptive effect of Kaempferol-3-O-rutinoside, isolated from the plant Ouratea fieldingiana, on adult zebrafish (Danio rerio). Biomed. Pharmacother..

[27] Yu S., Guo Q., Jia T., Zhang X., Guo D., Jia Y., Li J., Sun J. (2021). Mechanism of Action of Nicotiflorin from *Tricyrtis maculata* in the Treatment of Acute Myocardial Infarction: From Network Pharmacology to Experimental Pharmacology. Drug Des. Devel. Ther..

[28] Wang Y., Chen P., Tang C., Wang Y., Li Y., Zhang H. (2014). Antinociceptive and anti-inflammatory activities of extract and two isolated flavonoids of *Carthamus tinctorius* L.. J. Ethnopharmacol..

[29] Hwang D., Kang M.J., Kang C.W., Kim G.D. (2019). Kaempferol-3-*O*-*β*-rutinoside suppresses the inflammatory responses in lipopolysaccharide-stimulated RAW264.7 cells via the NF-κB and MAPK pathways. Int. J. Mol. Med..

[30] Ruan Y., Zhu X., Shen J., Chen H., Zhou G. (2024). Mechanism of Nicotiflorin in San-Ye-Qing rhizome for anti-inflammatory effect in ulcerative colitis. Phytomedicine.

