# Supplementary material for: Investigating the Anti-Inflammatory and Pain-Relieving Properties of Glaucium corniculatum (L.) Rud subsp. refractum (NAB) Cullen and Glaucium leiocarpum Boiss., Along with Their Active Compounds
Source: Molecules. 2026 Aug 4;31(15):2711. doi: 10.3390/molecules31152711 (PMC13468668; doi:10.3390/molecules31152711)
Supplement: Supplementary file 1 [file molecules-31-02711-s001.zip › molecules-4399556-supplementary.pdf]

- **HPLC chromatograms and UV spectra supporting the phytochemical characterization of *Glaucium leiocarpum* extracts, fractions, and isolated compounds.**

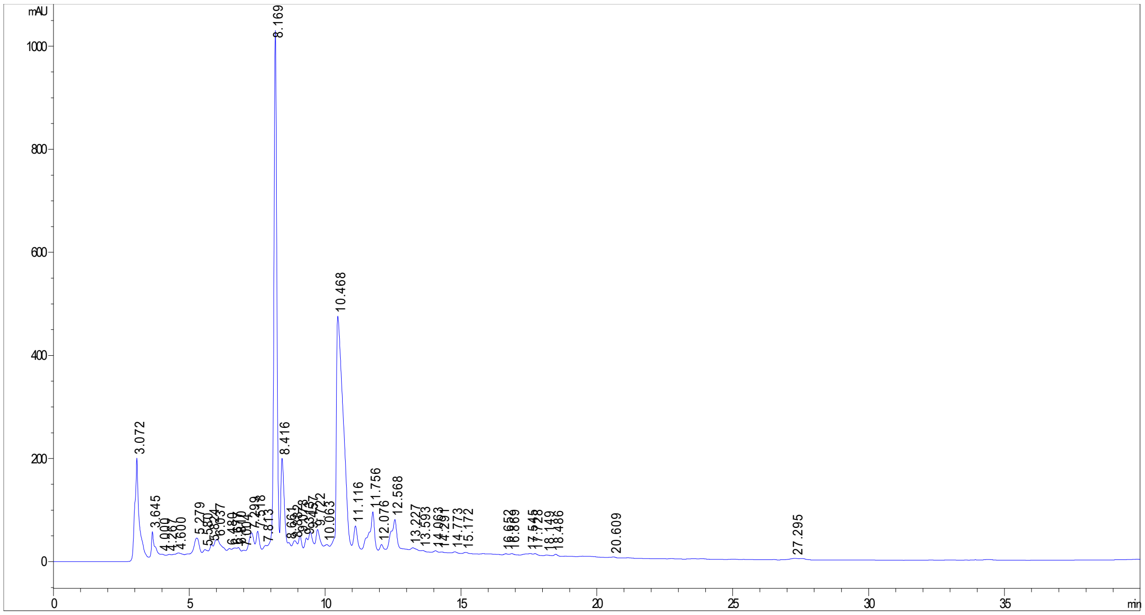

Figure S1. HPLC chromatogram (254 nm) of the total ethanolic extract of *Glaucium leiocarpum*.

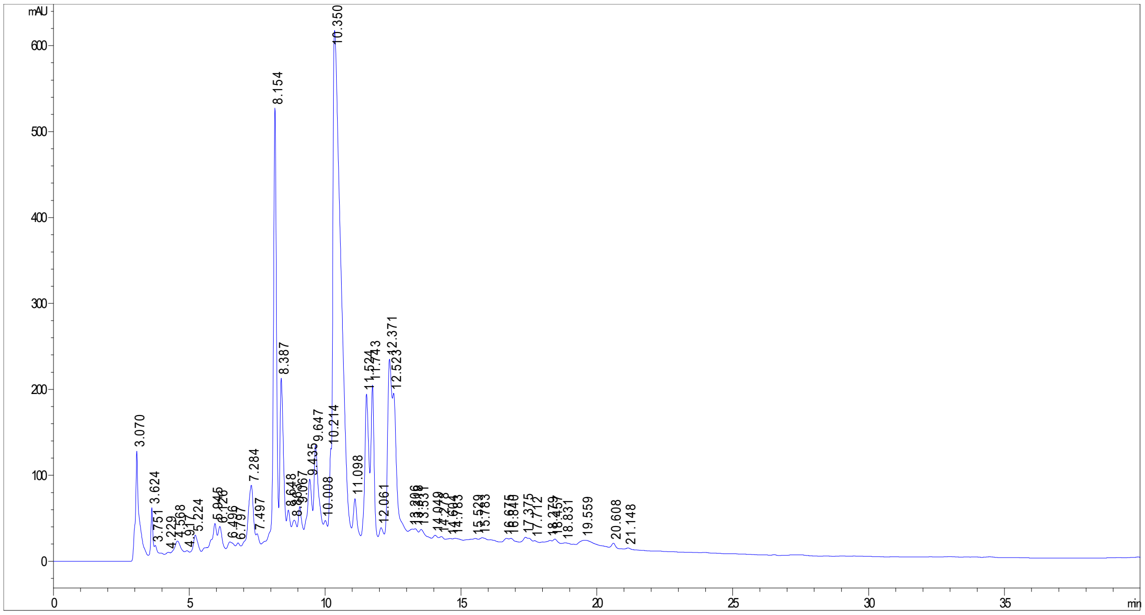

Figure S2. HPLC chromatogram (254 nm) of the ethyl acetate subextract of *Glaucium leiocarpum*.

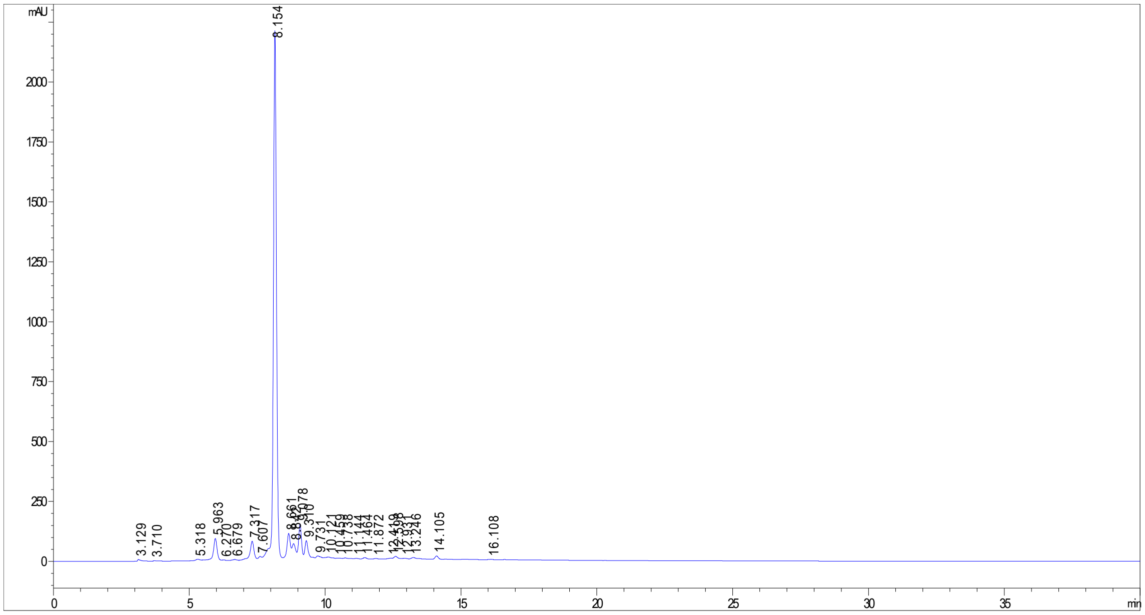

Figure S3. HPLC chromatogram (254 nm) of fraction A4 obtained from the ethyl acetate subextract of *Glaucium leiocarpum*.

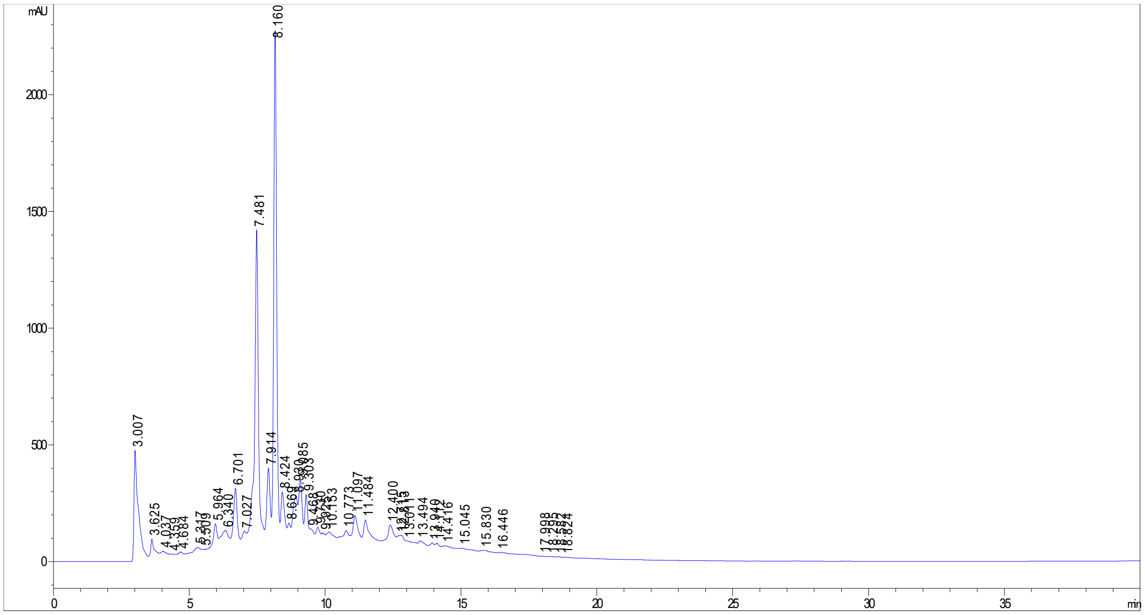

Figure S4. HPLC chromatogram (254 nm) of fraction Fr9–19 obtained from fraction A4 of the ethyl acetate subextract of *Glaucium leiocarpum*.

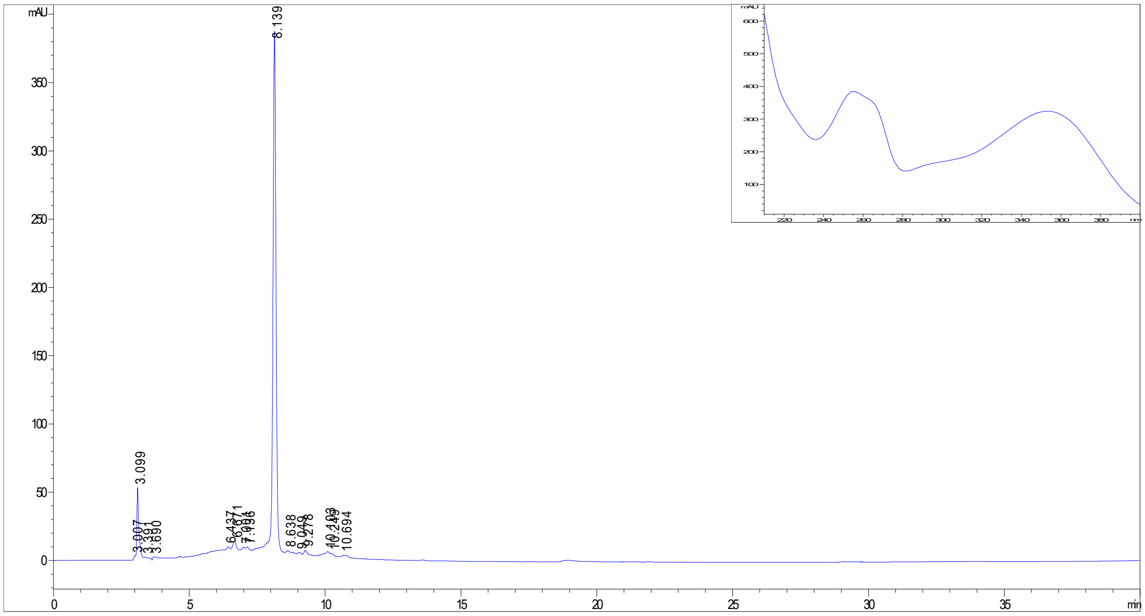

Figure S5. HPLC chromatogram (254 nm) and UV spectrum of rutin (Compound A).

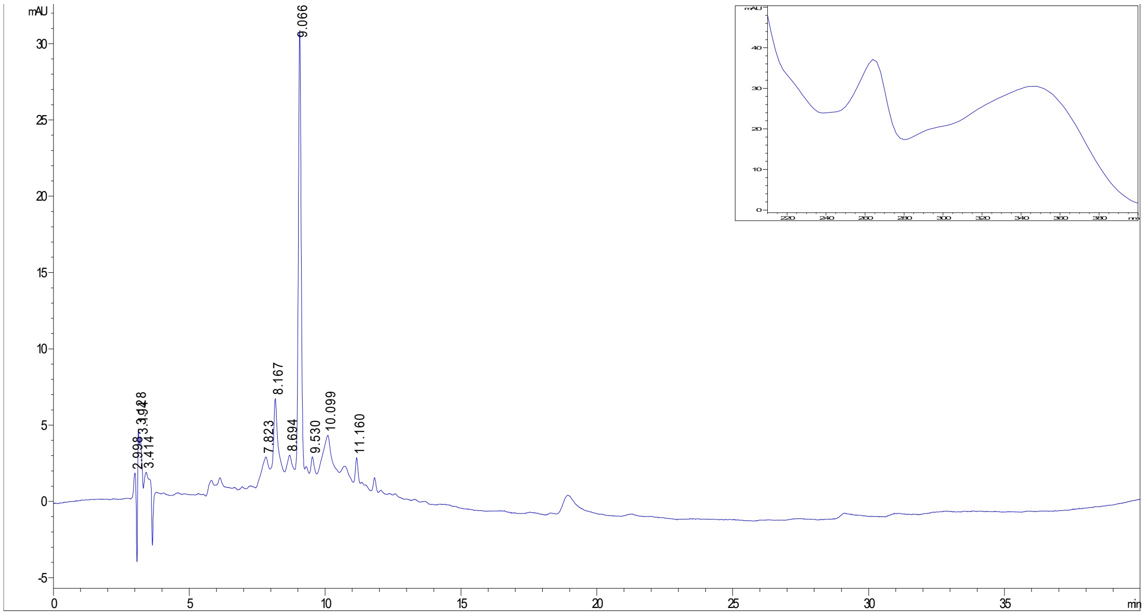

Figure S6. HPLC chromatogram (254 nm) and UV spectrum of nicotiflorin (Compound B).

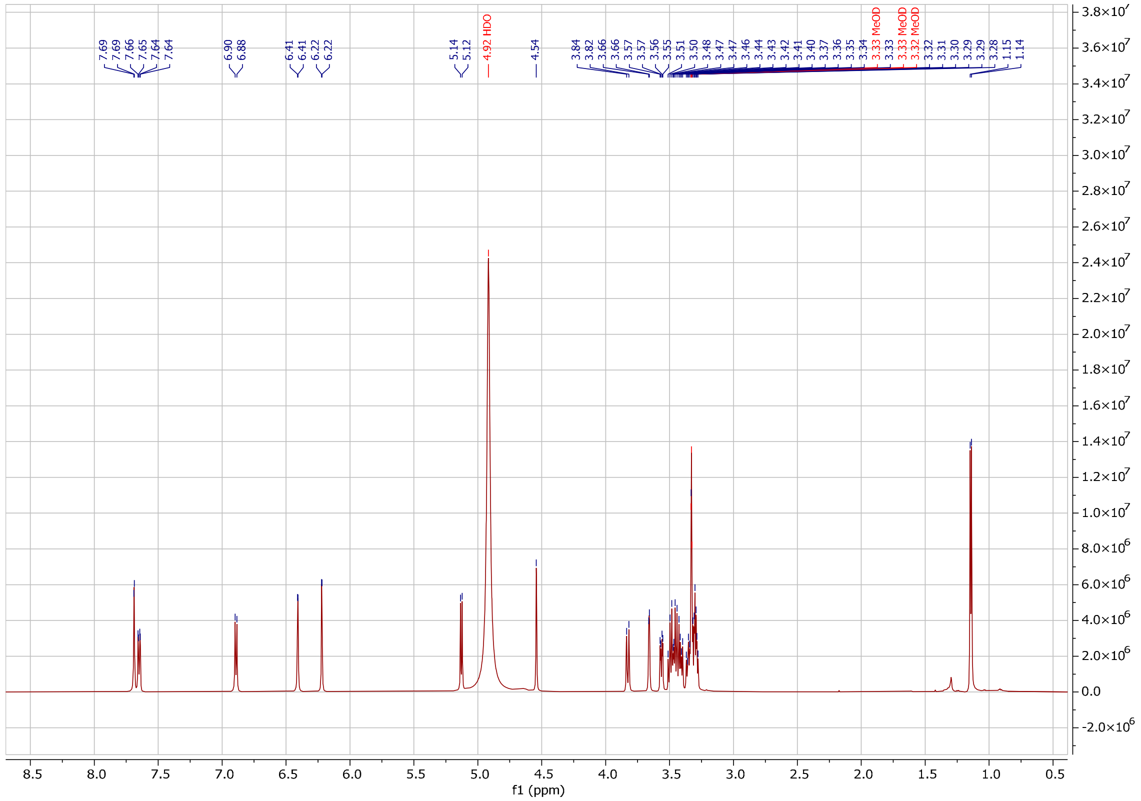

Figure S7. ^{1}H-NMR spectrum of rutin (Compound A).

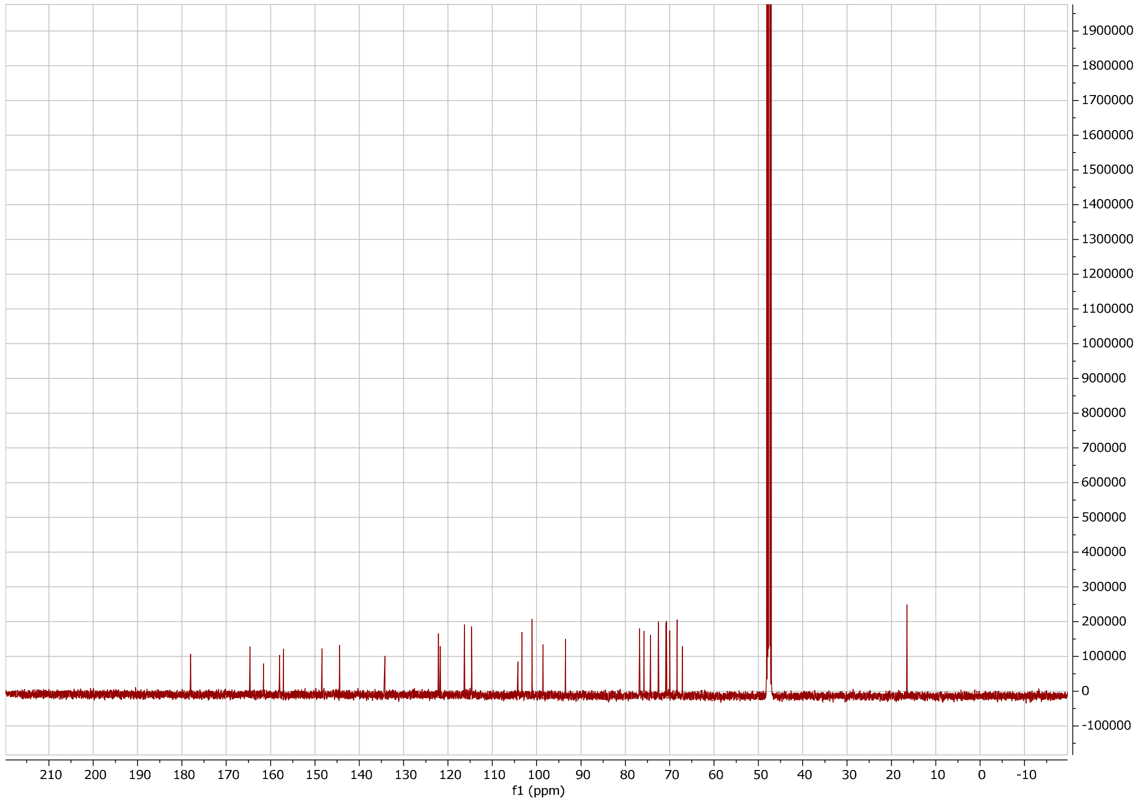

Figure S8. ^{13}C-NMR spectrum of rutin (Compound A).

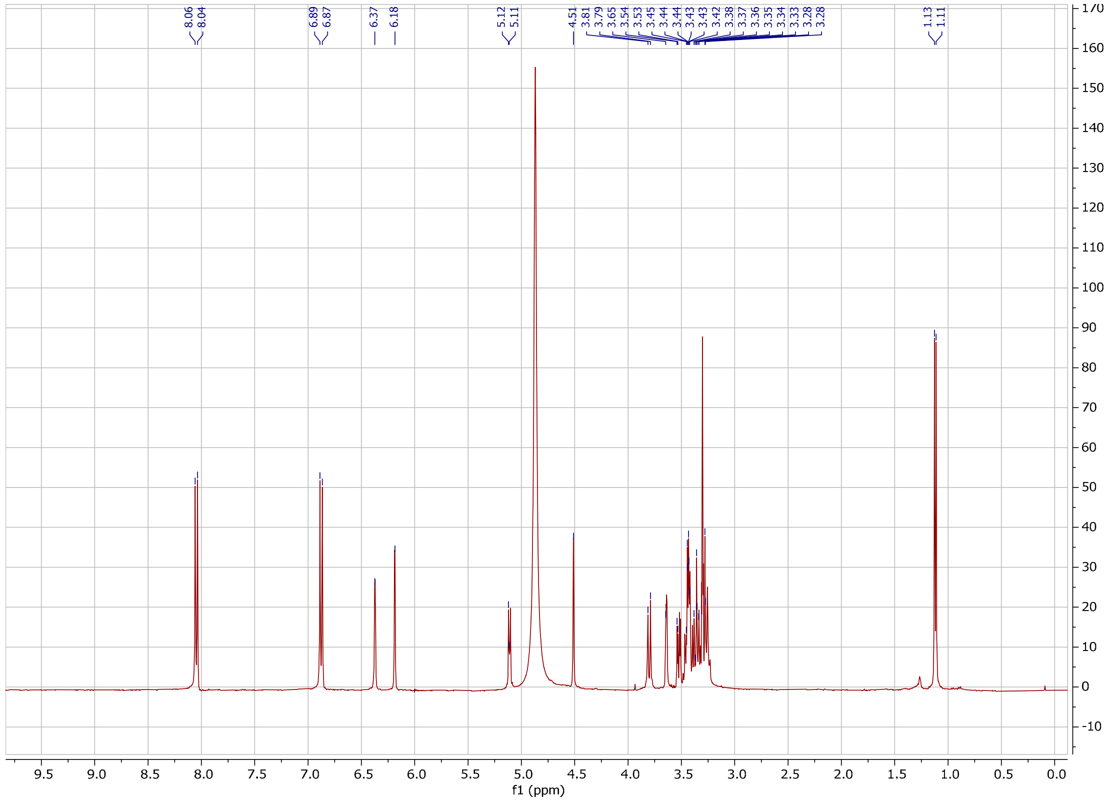

Figure S9. ^{1}H-NMR spectrum of nicotiflorin (Compound B).

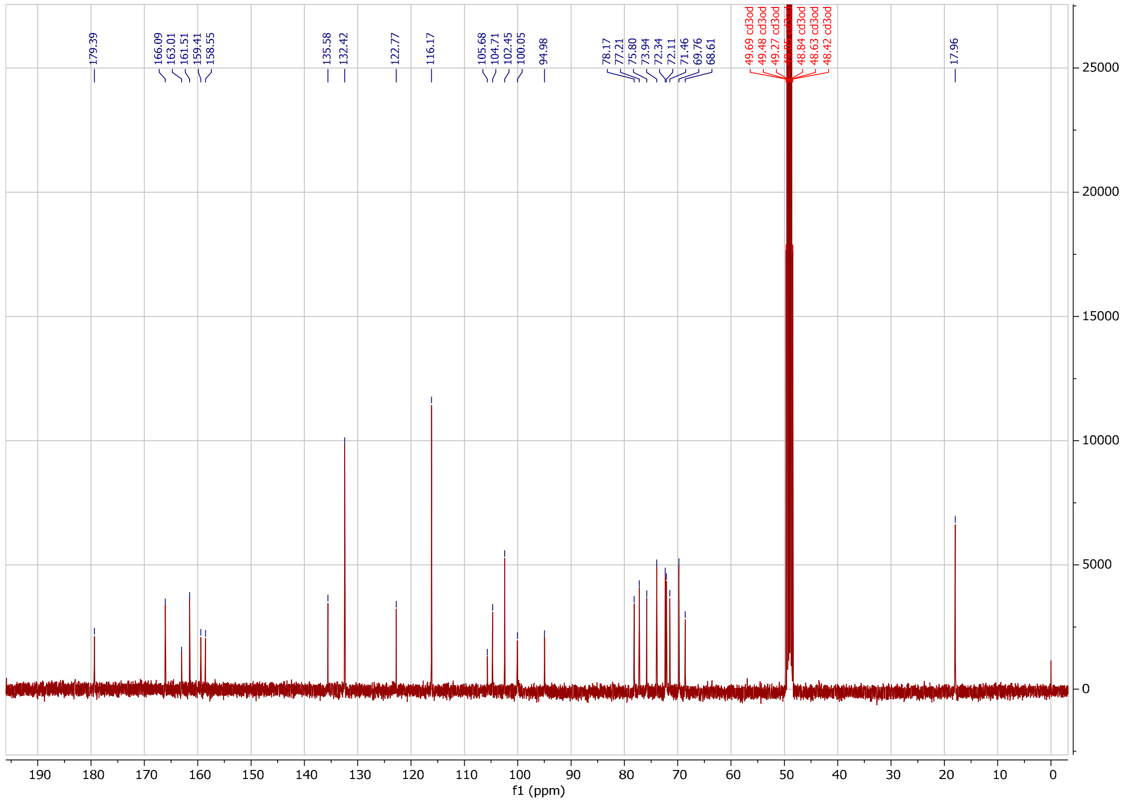

Figure S10. ^{13}C-NMR spectrum of nicotiflorin (Compound B).
